# AI-driven quality enhancement of medical datasets: a closed-loop framework of synthesis, annotation, quality control, and integration

**DOI:** 10.3389/fmed.2026.1947584

**Published:** 2026-10-01

**Authors:** Tao Tian, Siyuan Zhang, Xuchen Yang, Rui Shi

**Affiliations:** Department of IT & Data Management, West China Hospital, Sichuan University, Chengdu, China

**Keywords:** automated quality control, bias detection, causal reasoning, intelligent annotation, medical datasets, privacy-preserving computation, quality enhancement, synthetic data

## Abstract

The performance ceiling of medical AI is largely determined by the quality of its training datasets, yet traditional manual collection and annotation face a triple bottleneck of high cost, embedded bias, and limited scalability. This review takes “quality enhancement” as its unifying axis, integrating the four pillars of AI-driven medical dataset quality enhancement—synthetic data generation, intelligent annotation, automated quality control, and integration pipelines—and proposes a four-pillar integration framework as its core contribution. The framework organizes the four pillars into a closed loop: synthesis and annotation are coupled through dual-track feedback (a quantity track that replenishes data and a strategy track that adjusts generation strategy), while annotation and quality control are coupled through bidirectional verification. Beyond this forward enhancement loop, the framework further employs a bias amplification chain (as a heuristic insight) and the privacy–utility tradeoff as two tension lines, and incorporates Metrics Reloaded, FAIR, ALCOA+, and YY/T 1833 as sub-layer evaluation dimensions in a complementary manner. The review shows that AI-based enhancement methods have approached real data in statistical fidelity and downstream utility for select modalities, tasks, and evaluation settings, but systematic gaps remain in privacy, fairness, and clinical validity; single-pillar optimization is hypothesized, on the basis of indirect segmental evidence, to be insufficient to improve end-to-end quality, and closed-loop synergy is proposed as a testable proposition rather than an established requirement; integration pipelines are already usable for single-disease, single-modality settings, whereas cross-institutional, cross-modal, and cross-disease generalization remains the principal barrier, with federated construction and foundation-model-driven approaches as promising directions.

## 1 Introduction

### 1.1 The dependence of medical AI on high-quality datasets and current bottlenecks

The performance ceiling of medical artificial intelligence (medical AI) is largely determined by the quality of its training datasets. Topol (1) positions medical AI as “the convergence of human and artificial intelligence,” emphasizing that high-quality data is the prerequisite for this convergence. In recent years, deep learning and foundation models have achieved remarkable progress in medical image classification, lesion segmentation, and clinical text understanding, but these advances rely heavily on large-scale, high-quality, consistently annotated datasets—and the systematic preparation of medical imaging data for machine learning is itself a non-trivial engineering endeavor, systematically described by Willemink et al. (2).

Dataset bias directly degrades model performance: Larrazabal et al. (3) empirically demonstrated that gender imbalance in medical imaging datasets causes classifiers to perform persistently worse on the under-represented gender, revealing how deficiencies in the “representativeness” dimension propagate into model unfairness.

Constructing medical datasets, however, faces multiple bottlenecks. First, data hunger and the long-tail problem: rare diseases, minority populations, and edge cases are scarce in real data, causing model performance on these subgroups to drop sharply. Second, annotation scarcity and cost: medical annotation must be performed by qualified physicians, making the per-instance cost far higher than in general domains, and inter-annotator agreement is often low owing to differences in expert judgment. Third, privacy barriers: medical data are governed by regulations such as HIPAA, GDPR, and China's Data Security Law and Personal Information Protection Law, which restrict cross-institutional sharing and impede large-scale centralized dataset construction. Synthetic data augmentation has been shown to improve downstream performance: Frid-Adar et al. (4) used GANs to generate synthetic liver lesion images for augmenting CNN classification, empirically demonstrating the utility of synthetic data for downstream tasks and revealing a viable path of “trading quality for quantity.”

### 1.2 Cost, bias, and scalability issues of traditional manual collection and annotation

Traditional medical dataset construction, centered on manual collection and expert annotation, suffers from three structural problems. The cost problem: tasks such as annotating radiology reports and delineating pathology slides consume substantial physician hours, making the annotation cost of large-scale datasets high (industry estimates commonly place this at the million-dollar level, with annotation cycles often measured in years). The bias problem: manual collection is inherently constrained by single-institution, single-region, and single-population limits, producing selection bias and coverage bias; the landmark study by Obermeyer et al. (5) revealed that an algorithm using healthcare costs as a proxy for healthcare need led to systematic underestimation of Black patients, demonstrating that even “real” data can embed deep biases. Norori et al. (6) further categorized medical AI bias into data-acquisition bias, annotation bias, and algorithmic bias, and proposed systematic mitigation through open-science strategies, providing an acquisition–annotation–algorithmic (lifecycle-stage) taxonomy of bias, distinct from the mechanism-level types (selection, coverage, confounding) examined in §5.2. The scalability problem: manual workflows do not scale linearly with data volume, and inter-annotator inconsistency worsens as teams grow. Large language models (LLMs) have shown potential to replace human effort in text annotation: LLMs exhibit a significant annotation-efficiency advantage (7), but this advantage must be empirically re-examined in medical settings (see §4.1)—this both illustrates the opportunity of AI-driven enhancement and signals that the new risks it introduces (hallucination, domain shift) must be governed.

### 1.3 Technical enablers of AI-driven quality enhancement

Four classes of maturing technologies make AI-driven dataset quality enhancement possible. Generative models: generative adversarial networks (GANs) (8, 9) and diffusion models (10, 11) can now synthesize high-fidelity medical images and electronic health records (EHRs) (12, 13), while differentially private synthesis (14, 15) adds provable privacy guarantees—together providing means for long-tail completion and privacy protection; a review of cross-modal synthesis by diffusion models in medical imaging is given by Dayarathna et al. (16). Foundation-model-driven annotation: the medical image segmentation foundation model MedSAM (17), the general segmentation model SAM (18) (trained on the large-scale image–mask dataset SA-1B), and medical adapters (19) support prompt-guided zero-shot annotation, while CLIP (20) achieves image–text cross-modal alignment, substantially lowering the annotation barrier. Automated quality control: automated metric computation (21, 22) and bias detection (5) provide means for continuous quality control. Integration and orchestration: frameworks and tooling platforms such as nnU-Net (23), MONAI, and federated learning (24, 25) enable data collection, annotation, augmentation, quality control, and training to be orchestrated into reproducible automated pipelines. However, these technical enablers come with new risks: Shumailov et al. (26) showed that training on recursively generated synthetic data leads to irreversible “model collapse,” indicating that the self-consumption loop of synthetic data must be backed by quality control—this constitutes the practical motivation for the “enhancement–quality control” closed-loop argument. These four classes of technical enablers correspond respectively to the four pillars (synthesis, annotation, quality control, integration) and form the technical foundation of the four-pillar integration framework proposed in this review (Figure 1); the 2014–2025 evolution timeline of these four technology classes is shown in Figure 2.

**Figure 1:**
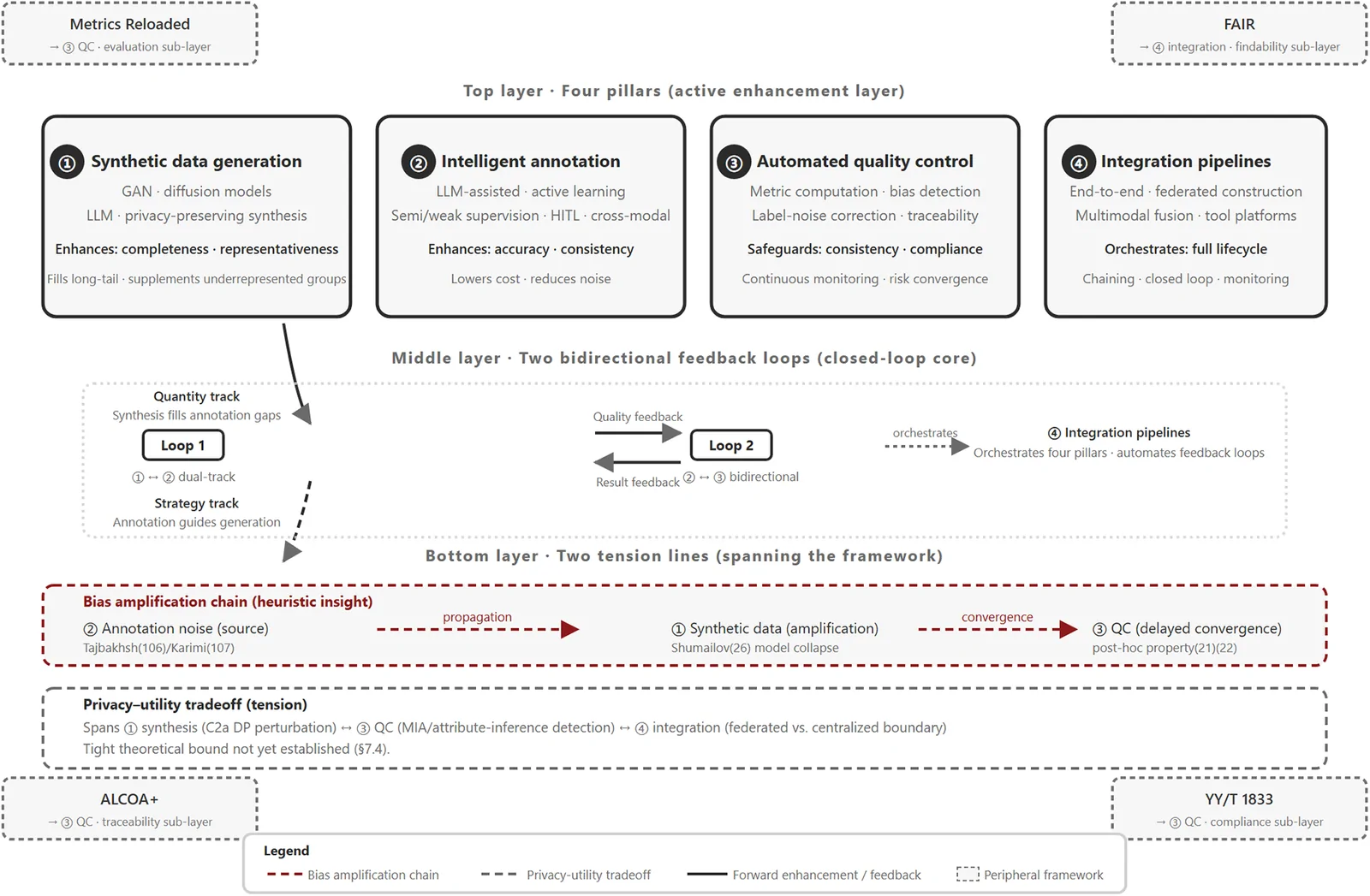
Four-pillar integration framework. The framework comprises four layers: (i) the four pillars juxtaposed—① Synthetic data generation, ② Intelligent annotation, ③ Automated quality control, ④ Integration pipelines; (ii) two bidirectional feedback loops—Loop 1 (① ↔ ②, with a quantity track and a strategy track) and Loop 2 (② ↔ ③); (iii) two tension lines—the bias amplification chain (②→①→③) and the privacy–utility tradeoff; and (iv) four peripherally embedded evaluation frameworks—Metrics Reloaded (49), FAIR (154), ALCOA+ (155), and YY/T 1833 (42)—as sub-layer dimensions. Dark-gray solid lines denote the forward enhancement flow and feedback loops (empirically supported relationships); a dark red dashed line denotes the bias amplification chain, and a gray dashed line denotes the privacy–utility tradeoff tension line—both conceptual relationships rather than empirically established mechanisms; bidirectional and unidirectional arrows denote feedback loops and track directions, respectively.

**Figure 2:**
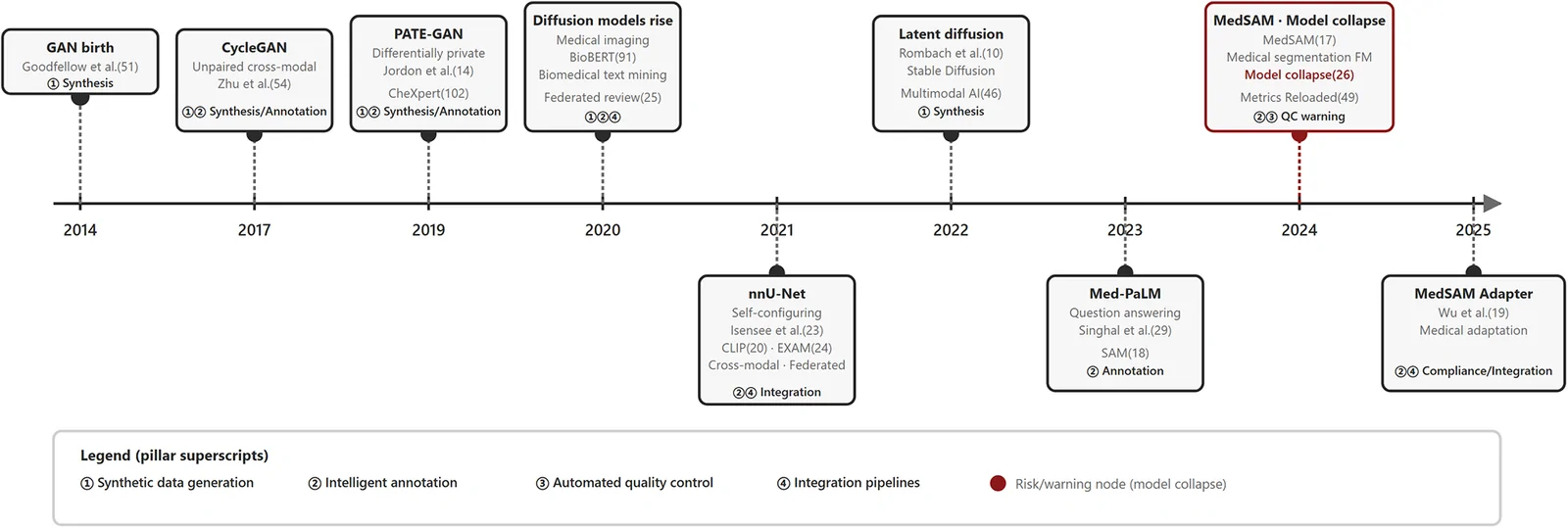
Timeline of AI-driven medical dataset quality enhancement technologies, 2014–2025. Key milestones span the birth of GANs (51) and CycleGAN (54) (2014–2017), differentially private synthesis (PATE-GAN (14)) and the chest-radiograph dataset CheXpert (102) (2019), the rise of diffusion models and biomedical pre-training (BioBERT (91)) alongside federated learning (25) (2020), cross-modal alignment (CLIP (20)), self-adaptive segmentation (nnU-Net (23)) and federated clinical prognosis (EXAM (24)) (2021), latent diffusion (10) and multimodal biomedical AI (46) (2022), medical LLMs (Med-PaLM (29)) and general segmentation (SAM (18)) (2023), the medical segmentation foundation model MedSAM (17), the model-collapse warning (26), and Metrics Reloaded (49) (2024), through medical foundation-model adapters (19) (2025).

### 1.4 Scope, contributions, and distinction from existing reviews

This review integrates the four pillars of AI-driven medical dataset quality enhancement—synthetic data generation, intelligent annotation, automated quality control, and integration pipelines—and proposes a four-pillar integration framework (Figure 1) as its core contribution. The research questions span the method spectra of the four pillars (RQ1), the efficacy and boundaries of each method along the fidelity/utility/privacy/fairness dimensions (RQ2), how existing quality frameworks are extended by AI methods (RQ3), and the cross-domain generalization maturity of end-to-end pipelines (RQ4).

The core contribution is a four-pillar integration framework that is an organizing synthesis of existing fragmented evidence rather than a wholly new theory awaiting empirical validation—its added value lies in making cross-pillar emergent relationships explicit. Within the framework, a bias amplification chain serves as a heuristic insight for organizing the discussion of risk rather than a necessary condition for the framework to hold (§7.6, §7.8).

To avoid overlap with existing reviews, this section delineates two boundaries:

(a) From Luo et al. 2025 (27): although “data quality enhancement” and “synthetic data generation” are literally named the same as two pillars here, the distinction is substantive—different objects (general LLM corpora vs. medical datasets bounded by clinical compliance and privacy), different closed-loop structures (an efficienc*y*-axis unidirectional flywheel vs. a feedback-bearing “enhancement–QC” loop argued by the medical-specific bias amplification chain), and different QC connotation (data selection vs. bias/label-noise/compliance/traceability). (b) From same-domain competing reviews: the foundation-models editorial of Wang et al. (28) uses foundation models as the organizing axis (model–data interaction), whereas this review uses dataset-quality-enhancement as the axis (four-pillar methods and their closed loop); specialty expert-consensus guidelines in Intelligent Medicine are modality-specific collection/annotation checklists, whereas this review is a cross-modal method review plus integration framework. (c) From single-pillar reviews (8, 9, 16, 25, 29) and the general data-centric-AI framework of Zha et al. (30): each covers a single pillar (or, for Zha et al., a general methodological framework without the medical multi-pillar closed loop and compliance constraints), whereas this review's added value is integrating the four pillars and explicitly characterizing their closed-loop quality-flow relationships (bias amplification chain, enhancement–QC coupling).

The above distinctions are compared across eleven columns in Table 1.

**Table 1:** Eleven-column comparison of this review with existing reviews.

Work (year)	Evid. basis	Organiz. axis	① Synthesis	② Annotation	③ QC	④ Integration	Closed-loop	Bias/quality-flow	Clinical–reg.	Eval. approach
Luo et al. 2025 (27) (LLM data-centric survey)	Method spectrum + framework	"Data value flywheel” for general LLM corpora	LLM only	No	Partial (data quality for training)	No	No	No	No	No
Wang et al. 2025 (28) (MedIA foundation-model editorial)	Editorial synthesis	Model–data interaction of medical foundation models	Partial (foundation models)	Partial	Not elaborated	No	No	No	No	No
Intelligent Medicine expert consensus	Expert consensus	Collection/annotation standards per modality	No	Partial (per-modality)	No	No	No	No	No	No
GAN medical imaging reviews (8, 9)	Method spectrum	GAN methods in medical imaging	GAN only	No	No	No	No	No	No	Yes (fidelity metrics)
Diffusion cross-modal review (16)	Method spectrum	MRI/CT/PET synthesis	Diffusion only	No	No	No	No	No	No	Yes (fidelity metrics)
LLM medical research (29) (Med-PaLM)	Model report	LLM clinical capability (QA)	LLM text	Partial (assist)	No	No	No	No	Partial	No
Federated learning medical review (25)	Method spectrum	Federated training optimization	No	No	No	Partial (training, not data construction)	Partial (collaboration)	No	Partial (privacy)	No
This review	Narrative synthesis (SANRA)	Four-pillar closed loop of medical dataset quality enhancement	Full (GAN/diffusion/LLM/privacy)	Full (LLM/AL/SS/WS/VLM/HITL)	Full (metrics/bias/noise/audit/monitor/compliance)	Full (federated/multimodal/tools/cases)	Yes—Loop 1 synthesis↔annotation, Loop 2 annotation↔QC, Loop 3 deployment→upstream (proposed)	Yes—bias amplification chain, QC-induced selection bias	Yes—§5.6 compliance, layer distinction (dataset ≠ device approval)	Yes—evidence matrix (Table 3), fidelity-validity layering

## 2 The quality enhancement framework

### 2.1 A multi-dimensional definition of medical dataset quality

Medical dataset quality is not a single scalar but a composite, multi-dimensional attribute. Synthesizing the five-dimensional EHR data quality model of Weiskopf and Weng (21) (completeness, correctness, concordance, plausibility, currency) and the fit-for-use framework of Kahn et al. (22), and incorporating dimensions newly salient in the AI era, this review adopts seven quality dimensions:

1. Accuracy: the degree of agreement between data values and the true clinical state;
2. Completeness: the extent to which required fields/records are present;
3. Representativeness: the degree to which the dataset covers the target population and its subgroups;
4. Fairness: the balance of performance across subgroups by race, gender, age, and socioeconomic status;
5. Privacy: the risk of the data leaking individual identities;
6. Timeliness: the degree to which the data are up to date relative to current clinical practice;
7. Consistency: the logical self-coherence across records, sources, and time

Quality-construct layering. These seven dimensions operationalize quality at the data and dataset levels, yet “medical dataset quality” is often conflated with non-equivalent downstream constructs. To prevent this conflation, the seven quality dimensions are situated within a distinct seven-layer construct hierarchy: (i) data quality—properties of individual records (accuracy, completeness, consistency, timeliness); (ii) dataset representativeness—coverage of the target population and its subgroups; (iii) model validity—the learning performance of a model trained on the data; (iv) clinical performance—the utility of that model on real clinical tasks; (v) fairness; (vi) privacy; and (vii) regulatory compliance—conformance to external standards and requirements. The seven quality dimensions operationalize levels (i), (ii), (v), and (vi); fairness (v) and privacy (vi) thus function both as constituent dimensions and as independent layers, being simultaneously dataset attributes and stand-alone evaluation axes. Note that fairness and privacy occupy different numbered positions in the two lists, because the dimensions enumerate data-level attributes whereas the layers span data to regulation. Their relationships are non-trivial: data quality (i) is foundational but not sufficient for any higher level; representativeness (ii) conditions both fairness (v) and generalization to clinical performance (iv); and model validity (iii) does not entail clinical performance (iv)—a model may learn the training distribution yet fail on clinical tasks. Regulatory compliance (vii) is an external constraint layer rather than an intrinsic data property. On this layering, the “quality enhancement” addressed by the four pillars acts on levels (i), (ii), (v), (vi), and (vii); model validity (iii) and clinical performance (iv) are downstream beneficiaries rather than direct objects of enhancement.

These seven dimensions are directly tied to the core tensions of AI-based enhancement: synthetic data improves completeness and representativeness but may harm privacy and fairness; intelligent annotation improves accuracy but may introduce annotation noise; automated quality control aims to monitor consistency and timeliness but often lags behind data production. The trade-offs among dimensions are the fundamental motivation for coordinating the four pillars rather than optimizing each in isolation; whether such coordination yields closed-loop synergy is a testable proposition (C1, §2.3 and §7.6), not an established conclusion.

### 2.2 Comparison of existing quality frameworks

Four mainstream frameworks currently exist in the domain of medical dataset quality, each with its own emphasis and blind spots. Table 2 provides a cross-comparison of the four frameworks:

**Table 2:** Comparison of existing medical dataset quality frameworks.

Framework	Source	Normative nature	Emphasis	Evaluation method	Blind spot	Scope	Pillar mapping	New capability after integration (C3)*
Metrics Reloaded (49)	Maier-Hein & Reinke, Nat Methods 2024	Research recommendation (community guideline)	Validation-metric selection	Metric taxonomy + checklist	Diagnosis-only; no generation	Image-analysis validation	③ QC evaluation sub-layer	Post-hoc metric → online QC monitoring signal (Loop 2)
FAIR (154)	Wilkinson, Sci Data 2016	Data-management principle (voluntary)	Findable/Accessible/Interoperable/Reusable (reusability)	4 principles, 15 indicators	Metadata-biased; no generation	General scientific data	④ Integration findability sub-layer	Retrieval after data exist → registration upon synthetic-data generation
ALCOA+ (155)	FDA/EU GCP	Data-integrity principle (regulatory guidance, GCP)	Data integrity + traceability (attributable, legible, contemporaneous, original, accurate + complete, consistent, enduring, available)	Attribute-by-attribute verification	Record-centric; no AI dimension	Clinical-trial data	③ QC traceability sub-layer	Record-level provenance → model-level traceability of synthesis/annotation
YY/T 1833 (42)	China NMPA	Industry standard (mandatory for NMPA registration)	AI-device dataset compliance	17 quality characteristics + audit	Compliance-biased; limited AI-enhancement coverage	AI-device registration (NMPA)	③ QC compliance sub-layer	One-time delivery review → continuous in-pipeline compliance monitoring

The asterisk (*) marks the “New capability after integration (C3)” column: the capabilities listed there are analytical projections of the integrated four-pillar framework, not empirically verified outcomes; conditions for their empirical realization are in §7.8. The “Normative nature” column signals that the four frameworks are not interchangeable: FAIR governs data reusability (findability/interoperability), ALCOA+ governs data integrity and traceability (attributable/contemporaneous/accurate records)—different objectives, neither substituting for the other. YY/T 1833 and ALCOA+ carry regulatory force (industry standard/GCP guidance); Metrics Reloaded and FAIR are voluntary community guidelines.

A common limitation of the four frameworks is that they are all primarily about “assessment/governance” and lack a unified organization for “how to use AI to actively enhance quality.” The systematic review by Hosseinzadeh et al. (31) on the dimensions, methods, and tools of medical data quality assessment corroborates this: existing medical data quality work focuses on assessment dimensions and audit tools, and the methodological organization of active AI-based enhancement remains a gap. The four-pillar framework does not claim to structurally merge the four frameworks (the four frameworks have heterogeneous evaluation objects; a genuine structural merger would require additional theoretical work); rather, it maps the four frameworks as sub-layer evaluation dimensions of the four pillars in a complementary organization—the four pillars provide the active-enhancement action layers of “generation/annotation/QC/orchestration,” while the four frameworks provide the evaluation yardsticks for each sub-layer.

The added value of integration (the C3 argument): the incremental value lies not in “an increase in the number of frameworks” but in the emergence of cross-pillar closed-loop behaviors that none of the four frameworks can accomplish alone. Under the original frameworks the four sub-layer yardsticks are static, post-hoc, and fragmented—each operating at a different time point, under a different responsible party, with no quality flow connecting them (Table 2). Once anchored to the same active-enhancement closed loop, these yardsticks shift from endpoint assessment to in-pipeline, real-time signals—metrics become Loop-2 feedback to annotation, FAIR registration moves to the moment of generation, ALCOA+  provenance extends to the model level, and YY/T 1833 controls become in-process checks. The last column of Table 2 itemizes the new capability each framework gains.

Asymmetry of mechanism maturity: the added-value mechanisms of Metrics Reloaded and YY/T 1833 are relatively mature (metrics shifted forward as online signals, compliance embedded as in-process checks), whereas FAIR and ALCOA+  require further operationalization—FAIR extended from “findability of existing data” to “registration upon generation,” ALCOA+ from “record-level provenance” to “model-level traceability"—both constrained by whether the generation pipeline embeds metadata registration and provenance infrastructure, so their mechanism maturity is asymmetric with the former two.

### 2.3 The AI-driven four-pillar integration framework

This review proposes an AI-driven four-pillar integration framework for medical dataset quality enhancement (Figure 1) as its core contribution. The framework is an organizing synthesis of existing fragmented evidence—rendering explicit and structuring the quality-flow relationships scattered across method spectra, so that the four-pillar closed loop, dual-track feedback, and other emergent relationships—invisible in any single-pillar review—become visible only once the four pillars are juxtaposed and organized. The added value of the framework lies precisely in making these cross-pillar emergent relationships explicit.

Framework nature declaration: the four-pillar integration framework is a conceptual, hypothesis-generating framework rather than a validated theoretical framework—its closed-loop synergy (C1) and the necessity of four pillars are propositions awaiting empirical testing, not established conclusions (§7.6; §7.8).

Positioning of “quality enhancement": distinct from data construction (producing data from scratch), data curation (curating existing data), and data augmentation (expanding data volume), quality enhancement emphasizes the active, multi-dimensional improvement and closed-loop optimization of existing or newly generated data—not only increasing volume but also improving fidelity, correcting bias, supplementing minorities, controlling privacy, and ensuring consistency.

The four pillars and their roles in the framework:

- Pillar ① Synthetic data generation: uses GANs (8, 9), diffusion models (10, 11), LLMs (12, 13), and privacy-preserving synthesis (14, 15) to generate high-fidelity data, filling long-tail and minority-group gaps and improving completeness and representativeness;
- Pillar ② Intelligent annotation: uses LLM-assisted annotation (7, 29), active learning (32, 33), semi-/weak-supervision (34, 35), human-in-the-loop (36), and cross-modal auto-annotation (17, 20) to reduce annotation cost and noise, improving accuracy and consistency;
- Pillar ③ Automated quality control: uses automated metric computation (21, 22), bias detection (5), label-noise correction (37, 38), provenance tracking (39–41), and compliance checking (42–44) to continuously monitor and correct quality, safeguarding consistency and compliance;
- Pillar ④ Integration pipelines: uses end-to-end workflows (23, 45), federated construction (24, 25), multimodal fusion (46), and tooling platforms (47, 48) to orchestrate the synergy of the four pillars and operationalize the full lifecycle.

Choice of the four pillars: the four pillars correspond to four quality-shaping functions—not to lifecycle stages. Each pillar performs a functionally distinct action on data quality: ① generates new data (filling gaps in volume and representation), ② assigns semantics (labeling and re-labeling), ③ verifies and corrects (detecting and remedying errors), and ④ orchestrates and lands (wiring the functions into an operational pipeline). These four functions provide a reasonable covering of the active quality-enhancement actions: any such activity creates data, labels it, checks it, or integrates it.

Against three pillars: the four functions are functionally distinct in the closed loop— merging ① and ② collapses Loop 1's dual-track feedback into a single-point self-loop (only separable “generating data” and “assigning semantics” permit dual-track quantity/strategy flows); merging ② and ③ fuses the noise source with its convergence node, dissolving the “propagation → amplification → convergence” chain and degrading QC into self-audit (losing independence); merging ③ and ④ lets the pipeline supervise itself, forfeiting QC's independent third-party monitoring of the integration layer (e.g., federated-construction drift, multimodal alignment error).

Against five pillars: the candidate stages outside the four functions—data collection, preprocessing, data governance, human oversight, validation, deployment, and monitoring—are lifecycle stages rather than quality-shaping functions, and are absorbed as the input conditions or output constraints of the four pillars rather than acting as independent enhancement layers. Collection and preprocessing provide input interfaces for the pillars (under ④); data governance and human oversight are boundary constraints on ②③ (under §5); validation is an output constraint on ③ [Metrics Reloaded (49) mapped to its evaluation sub-layer]; deployment and monitoring are operational contexts for ④ (under §6.6). Privacy-preserving synthesis (14, 15) is a sub-method of ① rather than an independent pillar. This four-way division is a reasonable organizational boundary chosen for functional clarity rather than a necessary partition, consistent with the framework's hypothesis-generating status.

The closed-loop property of the framework: the four pillars are not simply juxtaposed but form a closed loop with two bidirectional feedback loops—

- Loop 1: Synthesis ↔ Annotation dual-track feedback—this loop decomposes into two sub-flows:
- Quantity track (Synthesis → Annotation direction): synthetic data fills annotation gaps and balances the representation of minority groups, feeding back to the annotation pillar (emphasizing “quantity” replenishment);
- Strategy track (Annotation → Synthesis direction): the data gaps revealed by annotation-quality assessment guide the synthetic-data generation strategy (emphasizing “strategy” adjustment).

Loop 1 must be decomposed into the two sub-flows rather than a single bidirectional flow: the quantity track transmits data entities (gap-filling samples), driven by synthesis capability; the strategy track transmits meta-information about gaps (which subgroups or labels need filling), driven by annotation diagnosis. The two differ in object and trigger—if merged, “what to fill” and “why to fill” become conflated, and the attribution and operability of feedback are lost.

Loop 2: Annotation ↔ QC bidirectional feedback—annotation-quality signals feed back to QC, and QC results feed back to guide annotation optimization; the two perform bidirectional verification.

The two loops differ in nature: Loop 1 is a bidirectional coupling between synthesis and annotation (complementary quantity and strategy tracks flowing in opposite directions), while Loop 2 is a symmetric verification between annotation and QC, closer to bidirectional regulation. A proposed third path, Loop 3, extends this core—and the framework's closure—from deployment back to the upstream pillars (§6.6).

This closed-loop property organizes the known cross-pillar quality-flow relationships that existing single-pillar reviews (8, 9, 16, 25, 29) do not characterize. The embedded bias amplification chain (②→①→③) is positioned as a heuristic insight, juxtaposed with the forward enhancement loop to foreground the “enhancement–risk” duality; its segmented evidence, ablation conditions, and link to the C2a/C2b dual connotation are detailed in §7.6, Figure 3, and Table 3.

**Figure 3:**
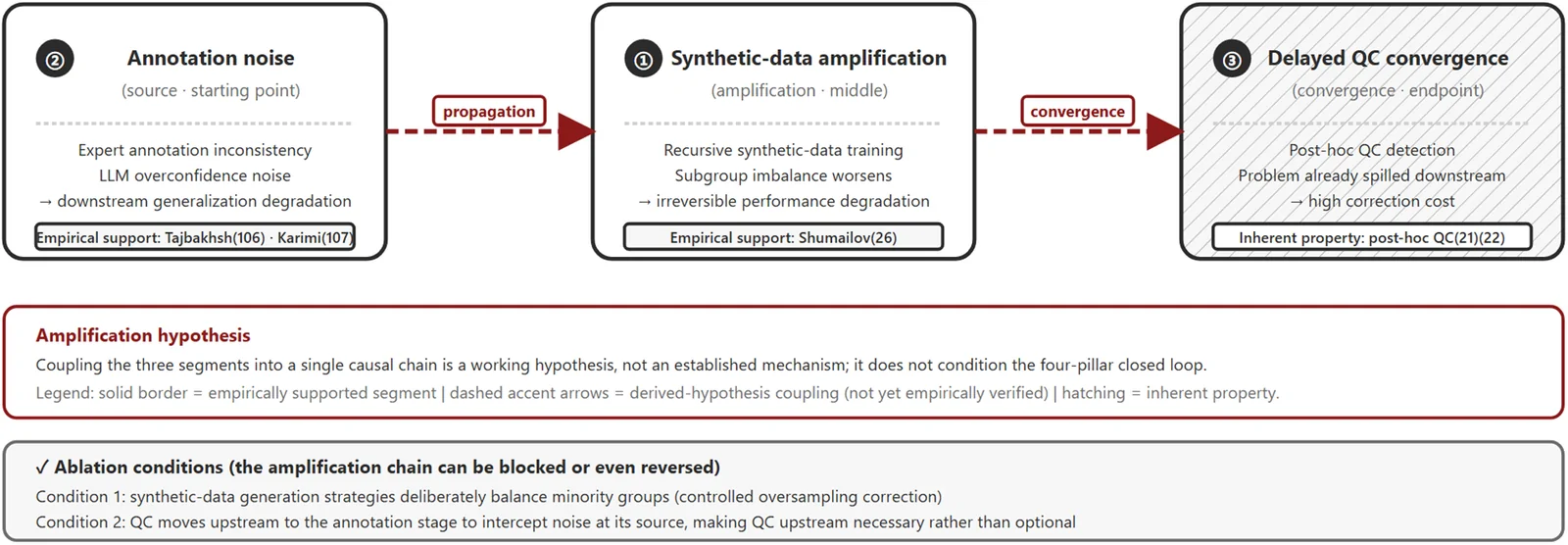
Bias amplification chain (heuristic insight). The three-segment path ② Annotation-noise source → ① Synthetic-data amplification → ③ Delayed QC convergence is shown with fill/border styles that encode the literature-support strength of each segment: Segment 1 (propagation, solid border = empirical support): annotation noise (106, 107) → downstream generalization degradation; Segment 2 (amplification, solid border = empirical support): recursive synthetic training (26) (model collapse) → irreversible degradation; Segment 3 (convergence, hatching = inherent property): post-hoc QC (21, 22) → high correction cost. Dashed coupling arrows between segments denote a derived hypothesis: the complete causal path linking the three segments into a single mechanism chain has no holistic empirical verification.

**Table 3:** Evidence matrix for the core arguments C1–C4.

Argument	Evidence type	Strength	Primary source	Principal gap
C1 Closed-loop synergy	Testable hypothesis	Indirect (segmented)	Integrative derivation: §3.6 (synthesis–QC), §5.3 (annotation–QC)	2⁴ factorial ablation across nine evaluation dimensions (Supplementary §S1.1)
C2a Privacy–utility tradeoff	Established finding (direction); testable hypothesis (tight bound)	Empirical, moderate	(14, 15, 50)	General tight theoretical bound (§7.4)
C2b Bias dual-edge	Established finding (amplification); author-derived (coupling conditions)	Mixed	(26); generation-strategy empirics	Coupling conditions for amplification vs. debiasing (§3.6, §7.6)
C3 Framework added value	Author-derived interpretation	Conceptual	The * capabilities in Table 2	Empirical verification of cross-pillar emergent capabilities
C4 Cross-domain generalization barrier	Direction established; across-case induction	Empirical, moderate (partly synthetic)	(24, 25, 46); §6.5 maturity gradient	Quantified generalization bounds across cases

Unified definition of the core arguments C1–C4: the framework is organized around four core arguments, uniformly defined as follows:

Testable corollary of C1: it is hypothesized that a pipeline containing synthesis + annotation + QC would outperform a synthesis + annotation pipeline without QC on representativeness (synthesis fills the long tail) and accuracy (QC corrects annotation noise), without degrading timeliness. As a proposition rather than an established result, this corollary can be evaluated with structured factorial ablation experiments on benchmark datasets (verification pathway in §7.6). However, synthetic data without QC backing may cause irreversible performance degradation (see the model-collapse discussion in §3.6), so the validity of C1 is premised on the presence of QC.

Table 3 classifies each argument by evidence type—established finding, author-derived interpretation, or testable hypothesis—together with strength, primary source, and principal gap. The bias amplification chain, a named proposition whose evidence is decomposed into established segments versus a proposed coupling, is detailed in §7.6 and Figure 3.

### 2.4 Mapping the framework to the dataset lifecycle

The four pillars do not act in isolation at a single moment but span the entire dataset lifecycle (Table 4):

**Table 4:** Mapping of the four pillars to the dataset lifecycle.

Lifecycle stage	Lead pillar	Supporting pillar
Collection	④ Integration (federated collection (24))	① Synthesis (gap filling)
Annotation	② Intelligent annotation	③ QC (consistency monitoring (101))
Enhancement	① Synthetic data	② Annotation (re-annotation of synthetic data)
Quality control	③ Automated QC	④ Integration (pipeline monitoring)
Deployment	④ Integration	③ QC (drift detection)
Monitoring	③ QC (continuous monitoring)	①② (feedback re-generation/re-annotation)

This mapping shows that single-pillar optimization is insufficient to improve end-to-end dataset quality—strengthening only synthesis (①) without QC (③) backing leaves model collapse (26) and privacy leakage (50) risks unintercepted; strengthening only annotation (②) without synthesis (①) gap-filling leaves the long-tail problem unresolved. The four pillars must be synergistic—the practical basis of the C1 closed-loop argument.

## 3 Synthetic data generation

### 3.1 GAN-based methods

Generative adversarial networks (GANs) (51) are a pioneering method family for medical synthetic data. An early breakthrough in training stability came from the Wasserstein GAN of Arjovsky et al. (52), which replaced the JS divergence with the Wasserstein distance to alleviate vanishing gradients, laying the foundation for stable training of subsequent medical GANs. Conditional GANs (cGAN/pix2pix) (53) achieve paired image translation through conditional inputs, widely used for segmentation-map-to-image and cross-modal conversion; CycleGAN (54) uses a cycle-consistency loss for unpaired cross-modal synthesis. These variants and their medical applications—MR↔CT translation (55, 56), multi-contrast MRI (57), low-dose CT denoising (58), and the general MedGAN framework (59)—are compared along three dimensions below.

This comparison reveals complementary rather than redundant positioning across three axes: pairing dependence (paired cGAN/pix2pix (53) and Dar (57) versus cycle-consistency CycleGAN (54) and DC-cycleGAN (56), the latter easing paired-data scarcity); cross-modal direction (structural MR↔CT translation (55, 56) versus same-modality dose-domain denoising (58); and enhancement objective (downstream-task input generation (55), image-quality restoration (58), or a general translation backbone (59)). Medical GANs thus differentiate along “paired → unpaired,” “cross-modal → same-modal enhancement,” and “specialized → general framework” rather than improving along a single performance line.

Frid-Adar et al. (4) generated synthetic liver-lesion images that augmented CNN classification, establishing that GAN synthesis can improve a downstream model beyond mere plausibility; Iqbal and Ali (60) applied GANs to fundus generation. The medical-GAN spectrum is surveyed by Yi et al. (9) and Kazeminia et al. (8), and Kebaili et al. (61) frame classical augmentation and GAN/diffusion synthesis as complementary along the fidelity–diversity tradeoff.

GANs carry defining failure conditions. Mode collapse drops low-frequency modes; since minority presentations are themselves low-frequency, collapse silently excises the long-tail cases most needing supplementation—converting a diversity failure into a representativeness bias (C2b) the QC pillar must audit. Training instability and paired-data dependence further constrain reliability, and on non-image modalities (tabular EHR, time-series) GANs recede before diffusion models and LLMs [Dayarathna et al. (16) treat both jointly]. Clinically, GAN-derived images may pass visual plausibility yet violate anatomical constraints, so acceptance hinges on demonstrated downstream benefit and bias audit, not realism—synthetic images are training data, not clinical evidence (§7.2).

### 3.2 Diffusion models

Diffusion models generate data through a progressive denoising process, outperforming GANs in fidelity and diversity, and have become the new mainstream for medical image synthesis (the synthetic-data method taxonomy is in Figure 4). Their methodological foundation was laid by three milestone works: the denoising diffusion probabilistic model (DDPM) of Ho et al. (62) established the “forward noising–reverse denoising” generation paradigm, Song et al. (63) unified the continuous-time diffusion framework with stochastic differential equations (SDEs), and the DDIM of Song et al. (64) used non-Markovian sampling to substantially accelerate inference while preserving generation quality. Building on these, the latent diffusion model (LDM/Stable Diffusion) of Rombach et al. (10) performs diffusion in latent space, substantially reducing computational cost, and has been widely transferred to medical image generation [Dayarathna et al. (16) surveyed cross-modal synthesis of MRI/CT/PET]; Imagen by Saharia et al. (11) demonstrated text-conditional text-to-image diffusion capability, laying the foundation for medical text-conditional image synthesis. In medical applications, Fu et al. (65) used an energy-guided diffusion model for CBCT-to-CT synthesis. The advantages of diffusion models are training stability, high sample diversity, and strong controllability; their limitations are slow inference and the absence of medical-specific evaluation criteria (see §3.5). Diffusion and GANs are complementary: diffusion targets fidelity and diversity, while GANs retain a place in speed-sensitive scenarios.

**Figure 4:**
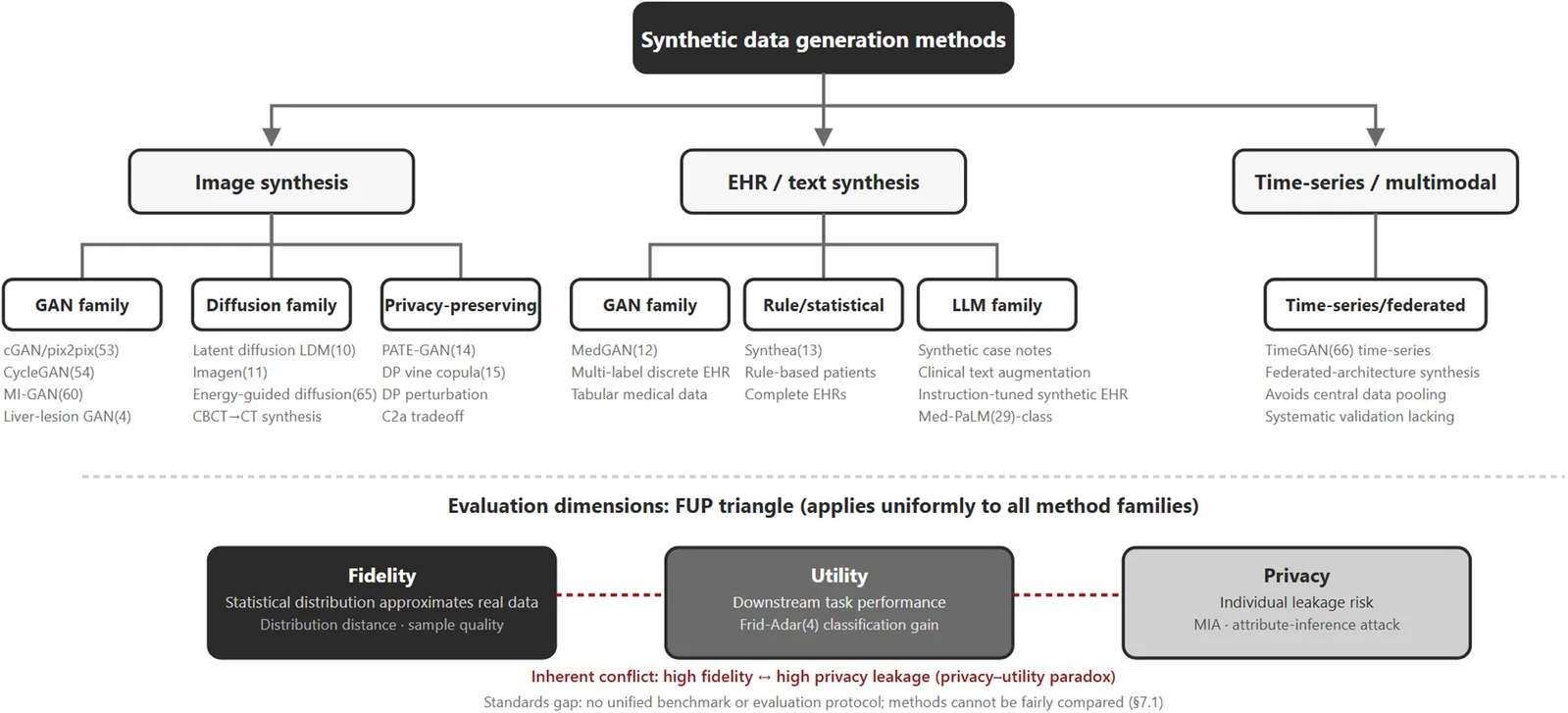
Synthetic-data method taxonomy tree. Organized by modality × method family—Image synthesis: GAN family: cGAN/pix2pix (53) (paired translation), CycleGAN (54) (unpaired translation), MI-GAN (60) (fundus), liver-lesion GAN (4) (augmenting classification); Diffusion family: latent diffusion LDM (10) (general), Imagen (11) (text-conditional), energy-guided diffusion (65) (CBCT→CT); Privacy-preserving family: PATE-GAN (14) (differentially private GAN), DP vine copula (15) (differentially private graphical-model synthesis). EHR/text synthesis: GAN family: MedGAN (12) (multi-label discrete EHR); Rule/statistical: Synthea (13) (rule-based synthetic-patient generation); LLM family: synthetic case notes, clinical-text augmentation, instruction-tuned synthetic EHR. Time-series/multimodal: time-series synthesis TimeGAN (66), federated synthesis.

LLM family: synthetic case notes, clinical-text augmentation, instruction-tuned synthetic EHR Time-series/multimodal: time-series synthesis TimeGAN (66), federated synthesis

### 3.3 LLM-generated synthetic medical text and EHR

For non-image modalities, tabular and EHR synthesis forms the foundation. Choi et al. (12) introduced MedGAN, the first GAN for multi-label discrete EHR records, while Walonoski et al. (13) developed Synthea, a rule-based synthetic-patient generator valued for controllability and compliance. Methodologically, CTGAN (67) became the de facto standard for mixed discrete/continuous tabular distributions, and TimeGAN (66) extended synthesis to temporal disease courses by combining autoregressive and adversarial training. Two reviews frame this space—Shickel et al. (68) on deep learning for EHR analysis and Ghosheh et al. (69) systematically comparing the fidelity and privacy of tabular generative models—and the MIMIC-III database (70) provides the widely adopted real-EHR benchmark for evaluation.

In recent years, LLMs have further expanded the capability boundary of synthetic medical text. In synthetic case notes, instruction-tuned LLMs [such as Med-PaLM (29)-class models] can generate structured case reports and narrative clinical notes, providing training corpora for natural-language-processing tasks—their core strategy is to fine-tune general LLMs on real case-note corpora so that the generated text preserves disease co-occurrence relationships and diagnostic-treatment logic. In clinical-text augmentation, LLMs can perform controlled augmentation of existing text through rewriting, synonym substitution, and entity replacement, improving the robustness of downstream NLP models on long-tail expressions. In synthetic EHR, the sequence-modeling capability of LLMs enables the generation of temporal case records and test/examination result sequences, with generation quality approaching real EHR distributions in statistical similarity. However, the empirical maturity of LLM-synthesized medical text is lower than that of tabular/EHR synthesis: tabular synthesis already has the systematic comparison of MedGAN, CTGAN, and other models by Ghosheh et al. (69) as an evaluation paradigm, whereas comparable evaluation of LLM-generated notes lacks equally systematic controlled studies—Yuan (71) integrates programmatic metrics, human evaluation, and LLM-as-judge into a comprehensive evaluation framework, suggesting the evaluation methodology is taking shape but complete end-to-end empirical evaluation remains future work.

The challenges of this direction are: the clinical fidelity of synthetic text is difficult to guarantee—LLMs may generate course descriptions that appear plausible but violate medical logic (e.g., incompatible drug combinations, inconsistent examination timing), a risk masked by their fluent linguistic capability, requiring domain-expert verification. Moreover, LLM hallucinations may conflate rare but real clinical presentations with atypical noise patterns, causing synthetic text to introduce spurious signals rather than real long-tail distributions. These challenges must be backed by the QC pillar (③) and human-in-the-loop.

### 3.4 Privacy-preserving synthetic data

Although synthetic data are regarded as a privacy-friendly solution, they are not inherently privacy-safe. Pilgram et al. (50) analyze synthetic-data generation (SDG) guidelines from the regulatory perspectives of the UK, Singapore, and South Korea, noting that synthetic data may still leak individual information and must be coupled with formal privacy guarantees. Zhang et al. (72) empirically assess the privacy risk of synthetic health data using membership inference attacks (MIA), finding that even with privacy-preserving generative models such as DPGAN, attackers can still infer training-set membership with high accuracy, corroborating that “synthetic ≠ private.”

Membership inference is one of several privacy threats against synthetic data; the broader spectrum also includes attribute inference, reconstruction, linkage and re-identification, memorization, model inversion, nearest-neighbor disclosure, and singling-out. A fundamental distinction separates two notions of protection: formal differential privacy (DP), a mathematically provable guarantee bounding any individual's information contribution regardless of adversary or auxiliary information; and empirical attack resistance, a measured resistance against a specific attack (typically MIA) that is not provable and degrades as attack methods evolve. The DP guarantees of PATE (73) and PATE-GAN (14) belong to the former; the MIA-based evaluation of Zhang et al. (72) and most empirical privacy assessments belong to the latter. Conflating the two—treating resistance to a current attack as a privacy guarantee—overstates the protection synthetic data provide.

Two approaches address privacy in synthesis: differentially private synthesis, where PATE (proposed by Papernot et al. (73)) achieves provable DP through teacher–student ensemble aggregation, PATE-GAN (14) couples this with GANs, and Gambs et al. (15) extend provably private synthesis beyond the GAN family via differentially private vine copulas; and federated synthesis, where raw data stay local and only parameters or synthetic products are exchanged, avoiding centralized aggregation risk (systematic validation of this direction remains insufficient; see §7.8).

The core tension of privacy-preserving synthesis is the privacy–utility tradeoff (C2a): the stronger the differential-privacy perturbation, the stricter the privacy guarantee, but the lower the utility of synthetic data. This tension is the technical-parameter manifestation of the C2 argument, and its theoretical tradeoff boundary has not yet been established (see §7.4).

### 3.5 Evaluation dimensions: fidelity, utility, privacy

Synthetic-data evaluation must account for three dimensions (the fidelity–utility–privacy, FUP, triangle): fidelity [statistical approximation to real data, with FID (74), precision/recall (75), and their mode-diagnosing improvement (76) as de facto standards]; utility (downstream-task performance of models trained on synthetic data, e.g., (4)); and privacy (real-individual leakage risk via MIA and attribute inference, operationally quantified by El Emam et al. (77), echoing the MIA-risk empirics of §3.4 (72)).

Fidelity–validity layering. The fidelity dimension above is commonly operationalized as statistical fidelity, but “fidelity” and “validity” are not equivalent and should not be used interchangeably. Six distinguishable layers intervene between synthetic data and real-world clinical value: (1) statistical fidelity—distributional similarity between synthetic and real data; (2) biological plausibility—conformance to known biological constraints and physiological coherence; (3) clinical validity—faithfulness to real clinical states and disease processes; (4) downstream model utility—performance of models trained on synthetic data on real tasks; (5) external validity—generalization across institutions, scanners, and populations; and (6) clinical decision utility—impact on clinical decisions and outcomes. The FUP triangle's “fidelity” refers only to layer (1). Claims that synthetic data “approach real data” are therefore bounded by statistical fidelity and do not, without independent evidence, extend to biological plausibility, clinical validity, or any higher layer. This bounding is particularly important for rare diseases and underrepresented populations (§3.6, §7.2): synthetic data may approach real data at the statistical layer while remaining clinically distorted, so synthetic representations must be validated at each successive layer rather than assumed valid by virtue of statistical similarity.

The FUP triangle has an inherent conflict: high fidelity often accompanies high privacy-leakage risk (the more synthetic data approximate the real, the more likely they are to reproduce real individuals)—an instance of the privacy–utility tradeoff (C2a). The principal problem of current evaluation is the absence of standards—different studies use different metrics, lacking unified benchmark datasets and evaluation protocols, making fair comparison among methods difficult (see §7.1).

### 3.6 Limitations: mode collapse, clinical fidelity, absent evaluation standards

The synthetic-data pillar faces three major limitations:

1. Mode collapse and model collapse: the mode collapse of GANs limits diversity; more severe is the model collapse proved by Shumailov et al. (26)—repeated training on recursively generated synthetic data causes irreversible performance degradation, and the self-consumption loop of synthetic data will self-destruct without QC backing (the amplification-segment path of the bias amplification chain is in Figure 3). This finding directly supports the “enhancement–QC” closed-loop argument: synthesis (①) must be accompanied by QC (③).
2. Insufficient clinical fidelity: synthetic images/text may be statistically plausible but clinically semantically erroneous (e.g., generating non-existent anatomical structures, temporal sequences violating physiological constraints); their clinical usability must be verified by domain experts, and prospective clinical validation of models trained on synthetic data is largely absent (see §7.2).
3. Absent evaluation standards: the FUP triangle lacks unified benchmarks, preventing fair comparison among methods (see §3.5, §7.1).

Regarding the bias dual-edge: synthetic data can both amplify bias (recursive training exacerbates subgroup imbalance) and debias (deliberate oversampling of minority groups, balancing representativeness). The conditional boundary between amplification and debiasing is not yet clear—depending on the generation strategy (whether controlled oversampling is applied) and QC synergy (whether subgroup representativeness is monitored). The two directions of the bias dual-edge (C2b)—amplification and debiasing—are a concentrated manifestation of the “non-neutrality” of synthetic data along the bias dimension; together with the privacy–utility tradeoff (C2a), they constitute the dual connotation of C2.

Synthetic bias amplification and subgroup-specific fidelity. Increasing the numerical representation of a minority subgroup does not necessarily improve its biological or clinical representation. Source-data bias is learned and reproduced by the generator: when the training data under-represent or stereotypically represent a subgroup, the synthetic data tend to replicate that distribution, and recursive training on synthetic output can amplify—rather than correct—the imbalance. Numerical oversampling may therefore produce subgroup-specific fidelity that is high at the aggregate statistical layer yet distorted at the biological-plausibility and clinical-validity layers (§3.5). To detect this, fidelity must be reported per subgroup (subgroup-specific fidelity) rather than as a single aggregate metric, and synthetic representations of minority subgroups require independent validation rather than acceptance by virtue of numerical increase. This amplification segment is the synthesis-stage link of the bias amplification chain (Figure 3); its downstream consequence—annotation noise amplified through synthesis and reaching QC only with delay—is developed in §4 and §7.6.

## 4 Intelligent annotation

Figure 5 — Comparison of intelligent annotation workflows. A comparison of four annotation modes—

**Figure 5:**
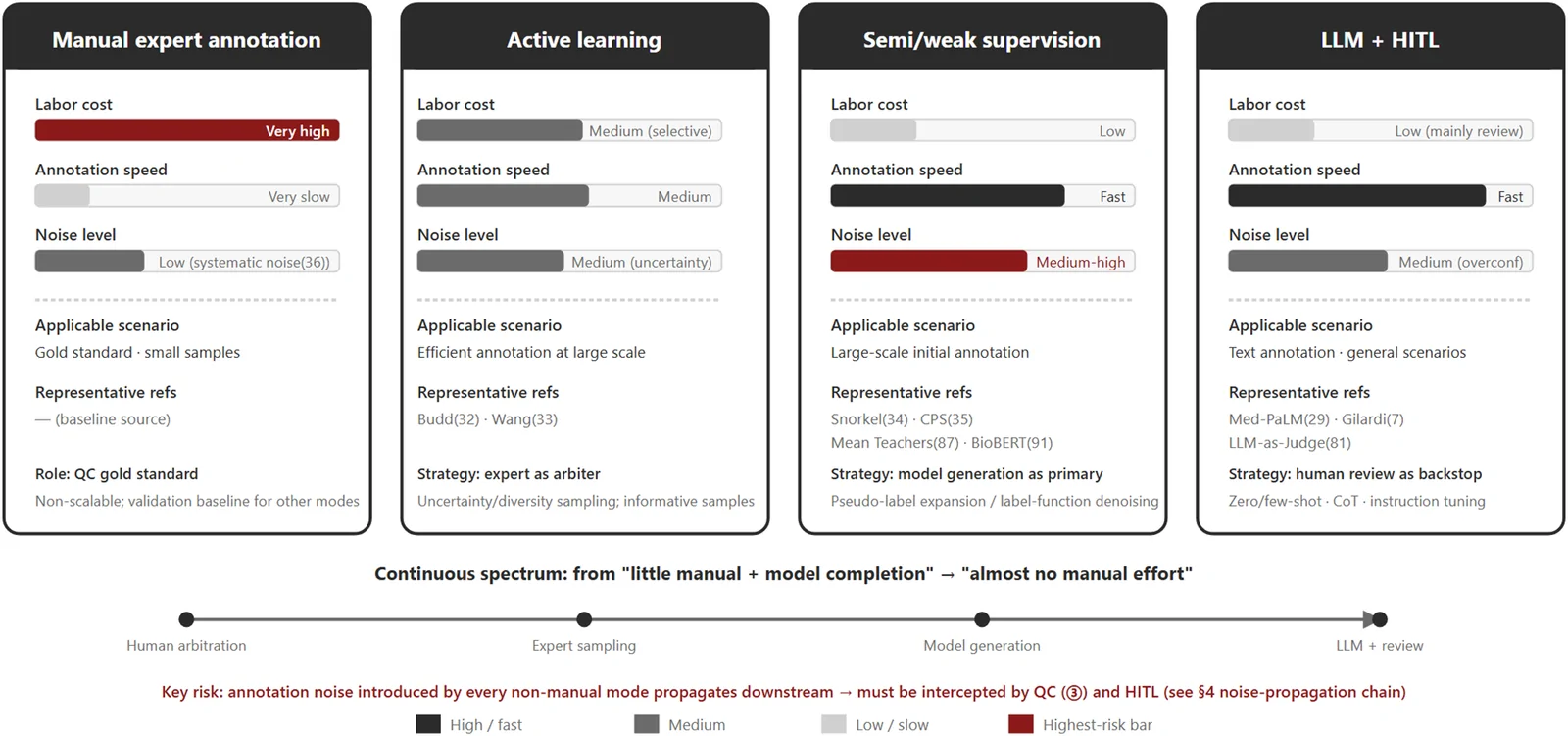
Comparison of intelligent annotation workflows. A comparison of four annotation modes—Manual expert annotation: very high labor cost, very slow annotation speed, low noise level (but systematic noise exists (36)); applicable to gold standards and small samples. Active learning: medium labor cost (selective annotation), medium speed, medium noise (depends on uncertainty estimation); applicable to efficient annotation of large samples (32, 33). Semi-/weak-supervision: low cost, fast, medium-high noise (depends on model quality); applicable to large-scale initial annotation (34, 35, 87). LLM + HITL: low cost (mainly review), fast, medium noise (overconfidence-noise risk (81)); applicable to text annotation and general scenarios (29, 7, 81).

### 4.1 LLM-assisted annotation

Intelligent annotation spans four modes—manual expert annotation, active learning, semi-/weak-supervision, and LLM + HITL—compared in Figure 5. LLMs bring a paradigm shift to medical data annotation. Med-PaLM by Singhal et al. (29) achieves expert-level medical question answering on the MultiMedQA benchmark through instruction tuning, establishing the foundation of LLM medical-annotation capability; the overall technical foundation, application spectrum, and challenges (hallucination, bias, privacy) of LLMs in medicine are systematically reviewed by Maity and Saikia (78). Gilardi et al. (7) empirically showed that ChatGPT's zero-shot text-annotation accuracy exceeds that of crowd workers by about 20% at about 1/30 the cost, demonstrating the significant advantage of LLMs in annotation efficiency; however, this study is based on general text-classification tasks, and when transferred to medical annotation—where higher professional accuracy is required and LLM hallucination risk is more critical—its accuracy advantage may shrink and must be empirically re-examined in medical scenarios. Medical-scenario empirics are accumulating: Holmes et al. (79) empirically evaluate the ability of foundation LLMs to relabel structure names per the AAPM TG-263 standard in radiotherapy, and Altalla et al. (80) compare the performance of generative LLMs on radiology-report annotation tasks—the two provide task-level empirics for LLM-assisted medical text annotation, while also corroborating that their accuracy is constrained by domain norms and requires expert review. The main forms of LLM-assisted annotation include: zero-/few-shot annotation (directly guided by prompts), instruction tuning (fine-tuning on medical corpora), and chain-of-thought (CoT, step-by-step reasoning annotation).

However, LLM annotation introduces new risks. The LLM-as-Judge study by Zheng et al. (81) reveals that LLM evaluation exhibits position bias, verbosity bias, and self-enhancement bias, suggesting that LLM annotation may produce overconfidence noise—i.e., erroneous annotations given by LLMs with high confidence, difficult to filter out by simple confidence thresholds. This risk means LLM annotation must be coupled with human-in-the-loop and QC (see §4.4, §5.3).

### 4.2 Active learning

Active learning reduces annotation cost by selectively querying the most informative samples for expert annotation. Two reviews by Budd et al. (32) and Wang et al. (33) systematically survey active-learning methods for medical imaging; the core query strategies include: uncertainty sampling (selecting samples about which the model is most uncertain), diversity sampling (selecting samples covering the feature space), and hybrid query strategies. The 2024 review by Wang et al. (33) further incorporates semi-supervised and self-supervised fusion strategies. At the level of general query algorithms, Sener and Savarese (82) model active learning as a core-set selection problem, characterizing representative sampling from a theoretical perspective; BADGE by Ash et al. (83) uses a joint diversity-and-uncertainty criterion based on gradient embeddings for batch selection, balancing information content and diversity and providing an efficient query strategy for large-scale annotation. For medical image segmentation, HAL-IA by Li et al. (84) embeds interactive annotation into an active-learning framework, and Gaillochet et al. (85) use stochastic batches to reduce the query cost of active segmentation—together advancing the engineering usability of active learning in medical settings.

Active learning recovers performance close to full annotation from a small expert-annotation budget, but it relies on the uncertainty estimation of the model's current state and may select the wrong samples when annotation noise is present.

### 4.3 Semi-supervised and weak-supervised annotation

To reduce dependence on large-scale annotation, semi-supervised and weak-supervised methods are widely used. Another path to lowering annotation cost is synthetic-data assistance: Vorontsov et al. (86) generate annotation-efficient synthetic images via image-to-image translation, demonstrating that synthesis-translation can significantly reduce manual delineation burden while preserving segmentation performance—reflecting the support of pillar ① synthesis for pillar ② annotation. Semi-supervision: Mean Teachers by Tarvainen and Valpola (87) improves semi-supervised learning with a weight-averaging consistency target; Cross Pseudo Supervision (CPS) by Chen et al. (35) achieves semi-supervised segmentation through consistency regularization, widely used in medical images. Representative works in medical semi-supervised segmentation unfold along the consistency-learning thread: UA-MT by Yu et al. (88) demonstrates the value of unlabeled data through uncertainty-guided self-ensembling on 3D left-atrium segmentation, the CNN-Transformer cross-teaching of Luo et al. (89) leverages heterogeneous architectures to improve semi-supervised segmentation robustness, and Luo et al. (90) further use uncertainty-rectified pyramid consistency (URPC) to mitigate pseudo-label noise—together establishing the dominance of consistency regularization in medical semi-supervised segmentation. Weak supervision: Snorkel by Ratner et al. (34) achieves weak-supervised annotation through programmatic labeling functions + generative-model denoising, a core method for medical text annotation. Domain pre-training: BioBERT by Lee et al. (91) is pre-trained on PubMed corpora, reducing the annotation demand for medical named-entity recognition and relation extraction; Alsentzer et al. (92) further release Clinical BERT embeddings on clinical-note corpora, bringing pre-training closer to the clinical language distribution.

The core difference between semi-supervised and active learning lies in the annotation strategy: active learning relies on expert selective annotation of uncertain samples, whereas semi-supervised learning uses the model itself to generate pseudo-labels to expand annotation—the former yields more reliable annotation but higher labor input, the latter is more automated but carries greater noise risk. The three form a continuum from “small manual + model completion” to “almost no manual,” with the choice depending on the task's tolerance for annotation inaccuracy.

### 4.4 Human-in-the-loop

The limitations of AI annotation make human-in-the-loop (HITL) indispensable. The study by Sylolypavan et al. (36) reveals that even high-level clinical experts exhibit systematic inconsistency in annotation, and this inconsistency, as “systematic noise,” materially affects the reliability of AI decisions—which in turn proves the necessity of HITL correction. The cascaded robust learning framework of Xue et al. (93) handles imperfect labels, combining robust loss with online label correction, embodying the engineering paradigm of HITL. At the interactive-segmentation level, Cellpose 2.0 by Pachitariu and Stringer (94) uses a “user makes minor corrections—model fine-tunes online” human-machine loop to enable non-experts to quickly train custom segmentation models. The core mechanisms of HITL include: expert consensus (multiple annotators reaching agreement), annotation-guideline automation, and inter-annotator agreement measurement (see §4.6).

### 4.5 Cross-modal auto-annotation

Foundation models enable cross-modal auto-annotation. CLIP (20) establishes zero-shot image–text alignment through contrastive pre-training, and SAM (18) achieves promptable zero-shot segmentation; its medical derivatives MedSAM (17) (1.57M image–mask pairs across 10 modalities and 30 + cancer types) and Medical SAM Adapter (19) adapt segmentation to medical scenarios. For image–text alignment, BiomedCLIP (95) (15M biomedical image–text pairs), the knowledge-enhanced EchoCLIP (96) and KAD (97) (echocardiography, chest radiography), and the multimodal multi-domain RADFM (98) directly serve cross-modal annotation. Flanders et al. (99) trace the resulting shift from manual delineation to model-generation-with-expert-review, and Liao et al. (100) show that report-generation's generation–verification loop can reverse-serve annotation.

Their differentiation follows two axes: modality coverage (general BiomedCLIP (95)/KAD (97) vs. echocardiography-specialized EchoCLIP (96) vs. multimodal RADFM (98) and knowledge enhancement (KAD (97)/EchoCLIP (96) inject explicit medical knowledge; BiomedCLIP (95)/RADFM (98) trade scale for capacity)—specialty precision versus general generalization in tension. The cited evidence (95–98) establishes general cross-modal capability, whereas annotation-task-specific benefit awaits dedicated investigation. Two failure mechanisms bear on clinical use: foundation models inherit biases from web-scale pre-training, so auto-labels can propagate under-representation into training corpora; and a single model labeling a corpus yields systematically correlated errors that defeat QC's premise—that annotation errors are mutually independent, uncorrelated noise—on which label-noise detection rests (§5). Auto-annotation is therefore a cost reducer requiring expert verification, not a replacement for gold-standard annotation—unverified auto-labels entering clinical training carry regulatory liability.

### 4.6 Annotation quality assessment

Annotation quality must be assessed by gold-standard comparison and agreement measures. Artstein and Poesio (101) systematically review inter-annotator agreement measures, including Cohen's *κ* (two annotators), Fleiss' *κ* (multiple annotators), and Krippendorff's *α* (with missing data and multiple types). The CheXpert dataset by Irvin et al. (102), with 224,000 chest radiographs containing uncertainty labels and accompanied by expert gold-standard comparison, is a benchmark for annotation-quality assessment and uncertainty modeling. Galbusera and Cina (103) review the methodology of radiology image annotation and data curation from the perspective of machine-learning practitioners, emphasizing the decisive role of annotation-quality control, toolchain selection, and curation workflow on downstream model performance. Agreement measures themselves are affected by annotator preference and random error: Liao et al. (104) model annotator preference and stochastic annotation error, revealing that systematic preference differences can distort agreement measures; Yang et al. (105) further evaluate inter-annotator agreement measures for medical image segmentation, noting that measure choice significantly affects the conclusion about “annotation quality.” Agreement measures are not only quality-assessment tools but also signals that trigger QC (pillar ③)—low-agreement regions should be flagged for review or synthetic completion.

### 4.7 Annotation noise propagating downstream (counterexample and risk chain)

The key risk of this pillar is the downstream propagation of annotation noise. The review by Tajbakhsh et al. (106) systematically surveys imperfect-dataset problems in medical image segmentation—annotation noise, small samples, incomplete annotation, domain shift; Karimi et al. (107) survey the techniques and remedies for medical image label noise, revealing the propagation mechanism of noise to downstream model generalization performance. This propagation chain constitutes the “source” risk in the four-pillar closed loop: annotation noise (②), if not intercepted by QC (③), will be amplified through synthetic data (①) (see the model-collapse discussion in §3.6), ultimately converging into model performance degradation. This makes forward-shifting governance to the annotation stage necessary. Across LLM-assisted, active-learning, and cross-modal annotation methods, validation remains confined to public-benchmark accuracy or segmentation metrics, with deployment evaluation in real clinical pipelines still insufficient.

The value of LLM annotation can effectively suppress overconfidence noise only when embedded in HITL and the QC closed loop—LLM annotation does not hold unconditionally; its cost-reduction and efficiency advantages depend on accompanying noise-governance mechanisms.

## 5 Automated quality control

### 5.1 Automated quality-metric computation

Automated quality-metric computation is the foundational layer of QC. The five-dimensional model of Weiskopf and Weng (21) (completeness/correctness/concordance/plausibility/currency) and the fit-for-use framework of Kahn et al. (22) provide the theoretical basis for metric definition; among them, completeness, plausibility (including consistency), and currency can be directly computed automatically. At the tool level, the ACHILLES (108) of the OHDSI ecosystem, together with data-quality dashboards (DQD), generates pre-computed summary statistics for EHR databases, enabling automated data-quality checking—Dixon et al. (109) report on the extended practice of this toolchain, and Hripcsak et al. (110) further elucidate how OHDSI supports reproducible conclusions from observational clinical data, together illustrating how the synergy of standardized data models and automated QC makes large-scale EHR quality auditing possible. ISO/IEC 25024:2015 (111) defines quantitative methods for data-quality measurement at the international-standard level. Automated metric computation converts manual spot-checks into continuous, full-coverage, reproducible monitoring, but its limitation is that it can only detect “known-pattern” anomalies and is powerless against semantic errors and bias.

### 5.2 Bias detection and mitigation

Bias detection is the higher-order capability of QC. The landmark study by Obermeyer et al. (5) revealed racial bias in medical algorithms—using healthcare costs as a proxy for healthcare need led to systematic underestimation of Black patients—becoming a classic case of selection bias and proxy bias. The ubiquity and harm of bias are further corroborated by two lines of work: Chen et al. (112) and Mhasawade et al. (113) survey algorithmic-fairness issues from the clinical-AI and public-health perspectives, noting that fairness risks pervade the entire chain of data collection, model training, and deployment; Seyyed-Kalantari et al. (114) empirically show that algorithms trained on chest radiographs of under-served populations exhibit systematic underdiagnosis bias, proving that subgroup-level bias can remain hidden even when overall performance meets the standard; Kim et al. (115) further propose transparent medical imaging AI via an image–text foundation model grounded in medical literature, enabling dataset bias to be detected through concept-level auditing.

The main types of bias include: selection bias (non-random sample selection), coverage bias (uneven subgroup coverage), and confounding bias (uncontrolled confounders). Detection methods include subgroup-performance-difference analysis, fairness metrics (e.g., equal-opportunity difference), and causal-analysis methods (116, 117). Jones et al. (117) systematically analyze medical imaging dataset bias from a causal perspective, proposing a causal-modeling framework to understand and mitigate bias; at the causal-methodology level, Feuerriegel et al. (118) survey causal machine-learning methodology for treatment-outcome prediction, and Richens et al. (119) prove that causal machine learning can improve the accuracy and generalizability of medical diagnosis—together providing a method toolbox for bias detection to move from statistical correlation to causal intervention. Mitigation strategies include resampling, reweighting, adversarial debiasing, and synthetic-data balancing (echoing the debiasing capability of pillar ①). The challenge of bias detection is that bias is often hidden in “seemingly normal” data and must be actively audited rather than passively discovered.

### 5.3 Label-noise detection and correction

Label noise is the principal risk introduced by intelligent annotation (pillar ②); its detection and correction is a key QC link. Song et al. (120) survey the landscape of noisy-label-learning methods for deep learning, providing a taxonomy for understanding noise types (symmetric/asymmetric/instance-dependent) and governance paradigms. Shi et al. (37) survey deep-learning methods for label noise in medical image analysis, proposing a unified taxonomy framework; Xue et al. (38) achieve robust medical image classification under noisy labels through global and local representation co-training. Common detection methods include confidence learning [Northcutt et al. (121) propose a systematic framework for estimating label noise], loss correction, and co-teaching [Han et al. (122) filter by small-loss samples to remain robust under extreme noise]. Correction strategies include automatic re-annotation, expert review (HITL backup), and synthetic-data replacement. This link is the core of Loop 2 (annotation ↔ QC bidirectional feedback)—label-noise detection results feed back to the annotation pillar to guide annotation-process optimization (annotation noise is the source in the bias amplification chain; see Figure 3).

### 5.4 Data auditing and provenance tracking

Data auditing and provenance tracking ensure the traceability and non-repudiation of datasets. MedRec by Azaria et al. (39) uses Ethereum smart contracts to implement blockchain-based medical-record access and permission management, laying the foundation for blockchain medical-data provenance; the systematic review by Elangovan et al. (40) confirms that the core applications of blockchain in healthcare include data integrity, logging, version control, and non-repudiation; Margheri et al. (123) further propose a decentralized medical-data provenance architecture based on the W3C PROV model, achieving traceable and tamper-proof provenance in cross-institutional data exchange. At the engineering level, data-version-management tools such as DVC (41) implement dataset version tagging and provenance based on Git. The value of provenance tracking is that when data-quality problems are discovered, they can be traced to specific source batches, annotators, and generation models, supporting root-cause analysis and accountability. Its challenges are the performance and scalability limitations of blockchain, and the overhead of version management on multimodal large datasets; moreover, provenance itself cannot defend against active attacks on data integrity—Finlayson et al. (124) reveal the adversarial-attack risk facing medical machine learning, suggesting that dataset QC must incorporate adversarial robustness rather than only auditing accidental errors.

### 5.5 Continuous-monitoring pipelines

Dataset quality is not achieved once but must be continuously monitored. Continuous-monitoring pipelines detect two types of drift: data drift (changes in input distribution) and concept drift (changes in input–output relationships). For example, imaging-equipment upgrades causing changes in image statistical characteristics, or clinical-guideline updates causing changes in diagnostic criteria, both require triggering dataset updates. Roschewitz et al. (125) empirically demonstrate automatic correction of performance drift under acquisition shift in mammography classification, showing that drift can be detected and partially mitigated after deployment—but full recovery still depends on dataset updates, and forward-shifting QC is superior to *post-hoc* correction. Continuous monitoring must be synergistic with the integration pipeline (pillar ④), embedding QC into end-to-end workflows to form a “monitor–alert–re-generate/re-annotate” closed loop. The other side of continuous monitoring is continual learning: the review by Qazi et al. (126) categorizes continual-learning methods and forgetting-mitigation strategies for medical imaging, indicating that datasets must be incrementally updated as tasks evolve, with QC preventing degradation of old knowledge. The FDA's Predetermined Change Control Plan (PCCP) (127, 128) requires AI medical devices to pre-declare and continuously monitor expected changes to models and data, reflecting the regulatory demand for continuous QC.

### 5.6 Automated compliance checking

Automated compliance checking is the regulatory dimension of QC. Three regulatory frameworks constitute the compliance baseline:

- YY/T 1833 (42): China NMPA's quality standard for AI medical device datasets; 1833.2 defines 17 quality characteristics and evaluation methods, and 1833.4 specifies traceability requirements;
- FDA GMLP (43): the ten Good Machine Learning Practice principles jointly issued by FDA/Health Canada/MHRA, covering multidisciplinary teams, data independence, clinical representativeness, model monitoring, and other full-lifecycle requirements;
- EU AI Act (44): EU Regulation 2024/1689 automatically classifies AI medical devices of class IIa and above as high-risk AI systems, imposing mandatory requirements on training-dataset quality.

Moreover, the regulatory pathway for LLM-class medical AI is also taking shape—Gilbert et al. (129) argue that LLM-driven AI chatbots should be approved as medical devices, suggesting that the compliance review of LLM-annotation/generation tools cannot remain outside medical-device regulation. The regulatory landscape is outlined by three works: Muehlematter et al. (130) analyze AI/ML medical-device approvals in the US and Europe (2015–2020), Vokinger et al. (131) track the FDA's action plan on continual learning in medical devices, and Vokinger and Gasser (132) discuss institutional differences in medical AI regulation between the US and Europe—together indicating that training-dataset quality is gradually moving from a soft requirement to a hard compliance constraint. Provided that checking items are clear and rules are encodable, automated compliance checking translates regulatory requirements into computable checks (e.g., traceability coverage, subgroup-representativeness attainment rate), turning compliance from “manual declaration” into “automated verification.”

A layer distinction must be drawn. Meeting a dataset-quality framework (FAIR/ALCOA+/YY/T 1833) is a data-layer condition, not equivalent to and not by itself securing the device-layer approval of an AI medical device (NMPA registration, FDA SaMD authorization, EU AI Act conformity assessment). The latter additionally requires model performance, risk management, clinical evaluation, and post-market surveillance (44, 127–132); an automated-compliance pass attests that a dataset satisfies a quality framework, not that a device is cleared for clinical use.

### 5.7 Quality control as the “convergence” node

Synthesizing the five sections, automated QC plays a “convergence” role in the four-pillar closed loop: annotation noise (② source) and synthetic-data risk (① amplification) are ultimately detected and corrected at the QC stage. However, current QC is mostly post-hoc—problems are discovered after data have been produced and models trained, when correction is costly and downstream effects may have occurred. This makes forward-shifting QC (embedding QC into the annotation and synthesis stages via Loop 1 and Loop 2) key to improving end-to-end quality. Yet automated detection covers known patterns while HITL backs up novel noise (overconfidence, model-collapse precursors)—the two divide labor, not substitute; the residual risk of fully automated QC cannot be ignored.

Forward-shifting QC introduces its own bias risk. Embedding QC earlier prevents error propagation but does not make QC bias-neutral: automated rules apply thresholds—outlier cutoffs, confidence filters, consistency checks—derived from a reference distribution, and using them as rejection gates can remove legitimate rare cases, minority-population observations, or clinically unusual presentations that fall outside the reference, introducing a QC-induced selection bias that narrows the dataset toward the majority and the expected. This is a double-edged property of forward-shifting QC: it blocks the bias amplification chain's error-propagation path (§7.6) yet may open a selection-bias path of its own. Mitigation requires that QC thresholds be validated against stratified subgroups rather than a pooled distribution—so a rule safe at the population level is not silently unsafe for a minority stratum—and that rejection decisions be logged and auditable, preserving removed cases as candidates for re-annotation (②) or re-synthesis (①) rather than permanent deletion. Without threshold validation and audit, forward-shifting QC risks trading one bias source for another.

Dataset quality and model performance are layered, not equivalent. The convergence above operates at the dataset-quality layer; it does not by itself establish model or clinical performance. Seven layers intervene between raw data and deployed clinical utility—dataset QC (§5.1–5.6), dataset validation (21, 22), model development, internal validation, external validation, clinical validation, and post-deployment monitoring (125–128)—each independent: passing one does not entail passing the next. A high-quality dataset is a necessary but not sufficient condition for a clinically valid model; model architecture, training, and deployment independently affect performance, and a model may pass internal yet fail external or clinical validation. This layering is downstream of—and distinct from—the quality-construct hierarchy in §2.1: the four pillars enhance dataset quality (the dataset-QC and dataset-validation layers) and, through forward-shifting QC, reach toward model development and internal validation, but the external, clinical, and post-deployment validation layers remain downstream concerns addressed by deployment-side governance rather than by dataset construction alone. Conflating dataset quality with model or clinical performance would overstate what the framework's QC convergence establishes.

Human oversight and accountability. Automated QC is not fully autonomous. Even where rules are encodable, clinical deployment retains human roles that machine checks cannot replace: expert spot-audits of flagged cases and of confidence thresholds themselves, adjudication of disputed labels, and ownership of rule and threshold settings. Accountability for a dataset therefore attaches to defined parties—the institution and the clinical/technical owners of the QC policy—rather than to the automation. This division mirrors the human-in-the-loop of pillar ② (§4.4): automation scales audit coverage, while humans arbitrate the novel cases automation cannot recognize (36, 129) and carry the regulatory accountability that no automated gate can assume (43, 44).

## 6 Integration pipelines

Pillar ④, the integration pipeline, is defined operationally as the cross-pillar orchestration layer that chains synthesis, annotation, and QC into a reproducible workflow and closes the feedback loops between them. Its common functional denominator is operationalization: federated learning, multimodal fusion, MLOps, automation, and version control are not equivalent techniques, but they share the function of turning isolated pillar activities into a governed, traceable, reproducible pipeline. Federated construction governs where data and computation reside; multimodal fusion governs how heterogeneous sources are combined; MLOps and automation govern how the pillars are chained and re-run; version control governs what was produced and when. These four sub-functions are grouped as one pillar because none of them generates data, assigns semantics, or verifies correctness directly—rather, each determines the conditions under which those pillar activities are executed, recorded, and repeated. Defining pillar ④ by this orchestration function, rather than by any single technique, is what lets it participate in closing the loop rather than merely enabling it (see §6.6).

### 6.1 End-to-end intelligent construction workflows

End-to-end intelligent construction workflows chain the four pillars into an automated data pipeline. The foundational deep-learning medical image analysis review by Litjens et al. (45) surveys the pipelines of core tasks such as classification, segmentation, and detection; nnU-Net by Isensee et al. (23) achieves self-adaptive configuration that automatically builds an optimal segmentation pipeline for any dataset without manual hyperparameter tuning, a benchmark method for automated pipeline construction. Self-supervised pre-training further reduces the pipeline's dependence on annotation volume: Yazdani et al. (133) use Swin UNETR self-supervised pre-training on PET/CT segmentation to achieve segmentation accuracy exceeding nnU-Net, demonstrating the value of self-supervision in automated segmentation pipelines. The core of end-to-end workflows is the extension of MLOps to the medical domain—orchestrating data collection, preprocessing, annotation, augmentation, QC, training, deployment, and monitoring into a reproducible automated chain that reduces manual intervention and variability, though its complexity also introduces an engineering barrier, making tooling platforms (§6.4) a necessary support.

### 6.2 Federated dataset construction

Federated dataset construction follows the “data stays put, models move” paradigm, enabling multiple institutions to collaboratively construct datasets without sharing raw data. The EXAM model by Dayan et al. (24) federated-training COVID-19 clinical-prognosis prediction across 20 global institutions, achieving AUC > 0.92, validating the feasibility of the federated paradigm on heterogeneous clinical data. The review by Rieke et al. (25) articulates the forward-looking vision of federated learning in digital health; Kaissis et al. (134) further combine secure multi-party computation with federated learning to achieve privacy-preserving collaborative training of medical imaging. The open-source infrastructure for federated medical imaging has also rapidly matured: Sheller et al. (135) early on validated multi-institutional federated training of brain-tumor segmentation without sharing patient data, the FeTS tool by Pati et al. (136) open-sources federated tumor segmentation, and OpenFL by Foley et al. (137) provides a general federated-learning library—together lowering the engineering barrier of federated dataset construction; on the federated-evaluation side, the MedPerf platform by Karargyris et al. (138) supports federated benchmarking of medical AI across institutions without sharing raw data, forming a closed loop of “federated construction–federated evaluation.”

Federated construction breaks through privacy barriers to achieve scale but is not itself a privacy guarantee: it is an architectural choice that reduces centralized exposure by keeping raw data local, without automatically ensuring privacy, security, or regulatory compliance. Five residual risks remain: non-IID data heterogeneity (cross-institutional distribution differences cause client-drift and may encode site-level bias that centralized training would average out); communication constraints (bandwidth and round limits can truncate convergence and degrade the final model); trust assumptions (a colluding or compromised coordinator can reconstruct or misuse local information); gradient leakage (exchanged gradients can leak information about the underlying training record under certain conditions); and aggregation attacks (gradient inversion can reconstruct private inputs from aggregated updates, so secure aggregation is an additional safeguard rather than a guaranteed property of the protocol itself). Federated learning is therefore not unconditionally superior to centralized training—when heterogeneity is severe, communication tightly constrained, or inter-institutional trust insufficient, federated may underperform—and a genuine privacy guarantee still requires complementary mechanisms such as differential privacy, secure aggregation, or secure multi-party computation (134); federated construction is privacy-enabling rather than privacy-guaranteeing, reflecting the fundamental barrier of cross-domain generalization (C4).

### 6.3 Multimodal data fusion pipelines

Multimodal fusion integrates multi-source data such as imaging, EHR, genomics, and wearables into a unified dataset. The multimodal biomedical AI review by Acosta et al. (46) systematically elaborates fusion strategies and architectural design, covering imaging–EHR, imaging–text, imaging–genomics combinations; the imaging–EHR deep-fusion direction is further refined by the systematic review of Huang et al. (139), classifying early/middle/late fusion strategies and providing implementation guidelines. In the imaging–genomics fusion direction, the PORPOISE framework by Chen et al. (140) integrates pathology histology and genomic data across 14 cancer types with multimodal deep learning for prognosis prediction, a representative work of pathology whole-slide-image (WSI)–genomics multimodal dataset construction. Beyond modality alignment, fusion validity is shaped by missingness mechanisms (MCAR/MAR/MNAR, of which MNAR silently encodes the absence of the hardest-to-represent cases) and four further risks—temporal misalignment, patient-level linkage errors, modality-specific measurement error, and information leakage (Supplementary §S2). Fusion pipelines must therefore be synergistic with QC: consistency checking of multimodal data is a new QC dimension, and the missingness mechanism itself should be diagnosed rather than assumed ignorable.

### 6.4 Tools and platforms

Tooling platforms are the engineering support of integration pipelines. Table 5 provides a comparison of the main tools:

**Table 5:** Comparison of tools and platforms for medical dataset quality enhancement.

Tool/Platform	Type	Applicable pillar	Core function	Modality	Open source
nnU-Net (23)	Segmentation framework	④ > ①	Self-adaptive segmentation-pipeline configuration	CT/MRI/PET	Yes
MONAI (156)	Medical imaging framework	④ > ① > ③	Full PyTorch pipeline: loading/preprocessing/training/evaluation	Medical imaging	Yes
MedSAM (17)	Segmentation foundation model	② > ①	Prompt-guided general medical segmentation (10 modalities)	Multimodal imaging	Yes
Label Studio (47)	Annotation platform	② > ③	Multi-type annotation with quality statistics	Image/text/audio/time-series	Yes
CVAT (48)	Annotation tool	② > ③	Image/video annotation with auto-annotation assistance	Image/video	Yes
ACHILLES (108)	Data-quality audit	③	OHDSI characterization/quality dashboard for EHR	Structured EHR	Yes
DVC (41)	Version management	④ > ③	Git-based dataset versioning + provenance	General data files	Yes
Snorkel (34)	Weak-supervision annotation	② > ③	Labeling functions + generative-model denoising	Text/image/tabular	Yes
SAM (18)	General segmentation model	②	Promptable zero-shot general segmentation	Natural images (adaptation for medical)	Yes
OHDSI toolchain (108)	Observational research	③ > ④	Standardized EHR data model + QC + analysis	Structured EHR	Yes

These tools cover the key links of the four pillars, but interoperability and standardization among them remain pain points.

### 6.5 Case studies: intelligent construction of lung-nodule, diabetic-retinopathy, and pathology WSI datasets

Lung nodule, diabetic retinopathy (DR), and pathology WSI are selected as three illustrative cases exemplifying the disease categories with the most mature public datasets and richest method spectra, forming a complexity gradient of “single-disease single-modal → multi-grade task → cross-disease multimodal.” The four-pillar participation and maturity of each case are summarized in Table 6.

- Lung nodule: LIDC-IDRI (141) (1,018 CT cases, four radiologists' annotations) and LUNA16 (142) (888 CTs, a detection challenge based on LIDC) are the standard benchmarks. Intelligent construction is fairly mature: synthesis completes rare nodule morphologies, active learning selects high-information slices, QC monitors annotator consistency, and the integration pipeline chains these into a reproducible process—four-pillar closed-loop maturity is highest under single-disease, single-modality (CT).
- Diabetic retinopathy: EyePACS (143) (approx. 35k–88k fundus images, 5-grade classification) and APTOS 2019 (144) (approx. 5,590 images) are the largest public DR datasets, whose method paradigm traces to Kermany et al. (145) on image-based deep learning for medical diagnosis. Intelligent construction emphasizes synthesis for grade-distribution balancing and LLM-assisted grading; but QC remains *post-hoc* consistency verification, and integration lacks a unified cross-dataset pipeline—maturity slightly lower than lung nodule.
- Pathology WSI: TCGA pan-cancer (146) (genomic and imaging multimodal across 33 cancer types) and MedMNIST/PathMNIST (147) (18 standardized 2D/3D datasets) are the foundation of pathology AI. Computational pathology faces quality challenges distinct from radiology and ophthalmology—scanner and staining variability, tissue-processing artifacts, multi-scale slide/tile relationships, slide-level versus region-level annotation, inter-observer variability, and diagnostic-label provenance—that make gigapixel-scale QC a distinct problem, not a minor extension of radiology QC (Supplementary §S3). MedMNIST/PathMNIST (147) supplies pre-tiled, pre-labeled patches at fixed resolution and so bypasses the slide/tile and scanner-variability problems that define true WSI construction; the two therefore represent substantially different construction and QC challenges (Supplementary §S3). The failure mechanism is site-bound: scanner- and staining-variability domain shift is biased by contributing institution, so a patch-level benchmark can hide slide-level bias that surfaces only at deployment; clinically, WSI-AI deployment requires stain normalization and multi-site validation, for which dataset-quality standards still lag. Intelligent construction emphasizes multimodal fusion and WSI segmentation annotation (SAM-class models); synthetic-data fidelity at gigapixel scale remains limited, and no cross-disease unified pipeline exists—the lowest maturity of the three cases.

**Table 6:** Four-pillar participation and maturity matrix for the three cases.

Case	① Synthesis	② Annotation	③ QC	④ Integration	Privacy	Fairness	External validation	Regulatory relevance	Modality/disease complexity	Closed-loop maturity
Lung nodule	Medium (rare morphologies)	Medium (active-learning slices)	Medium (annotator consistency)	Medium (reproducible pipeline)	Public de-identified CT benchmarks	Not systematically reported	Established via public benchmarks (LIDC/LUNA16)	Not systematically reported	Single-disease/single-modal (CT)	Higher
DR	Medium (grade balancing)	Medium (LLM-assisted grading)	Low (post-hoc consistency)	Low (no cross-dataset pipeline)	Public de-identified fundus benchmarks	Not systematically reported	Established via public benchmarks (EyePACS/APTOS)	Not systematically reported	Multi-grade/single-modal (fundus)	Medium
Pathology WSI	Low (gigapixel fidelity limited)	Medium (SAM-class segmentation)	Low (slide/tile consistency)	Medium (imaging–genomics fusion)	Multimodal linkage raises re-identification risk	Site-bound bias (scanner/staining variability)	Multi-site validation required; standards lag	Dataset-quality standards lag	Cross-disease/multimodal	Lower

Maturity criteria: Higher = all four pillars identifiable and ≥1 feedback loop operational with a reproducible pipeline; Medium = three pillars participating but feedback loops not automated or integration not cross-dataset; Lower = only single-point methods usable, feedback loops not closed, cross-domain unified pipeline not established. Each pillar's “Medium/Low” is judged by whether the method has public empirical implementation in that case. The four data-dimension columns (Privacy, Fairness, External validation, Regulatory relevance) are qualitative, based on dataset publicness, documented bias sources, benchmark-based validation practice, and dataset-quality-standard maturity; “Not systematically reported” marks dimensions not yet systematically addressed for that case, consistent with the illustrative (non-validating) status of these case studies.

The maturity gradient across the three cases supports the C4 argument: intelligent construction of single-disease, single-modality (lung nodule) approaches four-pillar closed-loop usability, multi-grade tasks (DR) are next, and cross-disease multimodal (pathology WSI) is still at the single-point method stage—a unified pipeline across diseases and modalities remains the principal barrier. “Four-pillar closed-loop usable” means all four pillars have public empirical implementation in that disease, at least one feedback loop is operational, and the construction process is reproducible (complete closed-loop-efficacy empirics are in §7.8 C1 boundary).

### 6.6 Integration pipelines as the orchestration layer

The integration pipeline (pillar ④) is not independent of the first three pillars but is their orchestration layer—it determines how the four pillars are chained, how the feedback loops are closed, and how QC is embedded. A well-designed integration pipeline should make the two feedback loops operate automatically: synthetic data fills annotation gaps (Loop 1 quantity track), QC results feed back to annotation optimization (Loop 2), and annotation-quality signals guide the synthesis strategy (Loop 1 strategy track). Current pipelines mostly remain at the “chaining” stage; automation of the feedback loops is still a future direction (see §7.7).

The distinction between an orchestration layer that merely enables the Loop 1/Loop 2 feedback and a genuinely four-pillar closed loop is whether pillar ④ itself generates feedback signals: orchestration alone routes signals originating inside pillars ①–③, whereas a closed loop additionally routes deployment-boundary signals back upstream. A closed four-pillar architecture requires pillar ④ to feed deployment-level evidence—performance drift detected in monitoring, calibration decay, subgroup performance gaps surfaced post-deployment, integration-level failures (e.g., a pipeline step that silently drops records), and detected distribution shift between the training environment and the live population—back into the upstream pillars: drift and distribution-shift signals can trigger re-synthesis (①) to regenerate under-represented strata; subgroup and calibration-decay signals can trigger re-annotation (②); and integration-level failures and QC-convergence anomalies can trigger QC adaptation (③) including re-tuning of the automated thresholds themselves (see §5.7). These deployment-to-upstream signals form a third feedback path—Loop 3—whose existence distinguishes a four-pillar closed-loop architecture from a three-pillar feedback system supported by an orchestration layer. This Loop 3 routing is a proposed property of a complete architecture rather than a feature demonstrated by current pipelines: today's systems implement monitoring and drift detection as operational practices, but the automated routing of deployment signals back into re-synthesis, re-annotation, and QC-adaptation decisions remains a design target, not an established capability (see §7.7).

## 7 Discussion: challenges, future directions, and limitations

### 7.1 Absent evaluation standardization and insufficient benchmark datasets

The evaluation of synthetic-data and annotation quality lacks unified standards. As described in §3.5, the FUP triangle has no unified benchmark, preventing fair comparison among methods; El Emam et al. (148) validate multiple utility measures across 30 health datasets for ranking consistency across synthetic-data generation methods, finding that measure choice significantly affects conclusions—corroborating that the absence of evaluation standards is the main cause of inter-method incomparability. Although annotation-quality assessment has agreement measures (101, 102), cross-dataset, cross-task benchmarks are still lacking. The future requires public synthetic-data and annotation-quality benchmarks so that methods are comparable and progress measurable.

### 7.2 Clinical trustworthiness and regulatory acceptance of synthetic data

The clinical usability of synthetic data is limited by its clinical fidelity (§3.6)—statistical plausibility does not equal clinical correctness. Eckardt et al. (149) survey the application prospects and clinical-translation pathways of AI-generated synthetic data from the perspective of cancer research and clinical trials, while noting that clinical trustworthiness and regulatory acceptance remain the principal bottlenecks for scaled application. At the regulatory level, FDA GMLP (43) and EU AI Act (44) impose requirements on training-data quality, but the regulatory pathway for synthetic data as training data is not yet fully defined. The future requires clinical-validation protocols and regulatory-acceptance frameworks that move synthetic data from a “research tool” to “clinically usable.”

### 7.3 Hallucination and domain shift in LLM annotation

The overconfidence noise (81) and domain shift (performance degradation of general LLMs in medical sub-domains) of LLM annotation are the core risks of the annotation pillar. The systematic review by Shool et al. (150) on LLM evaluation in clinical medicine shows that existing evaluations mostly focus on clinical question answering and decision-making capability, and the reliability evaluation of LLMs as annotators remains insufficient, with hallucination and domain-shift risks in annotation scenarios underestimated. Their downstream propagation (the noise-propagation chain in §4) makes forward-shifting governance necessary. Future directions include instruction tuning of medical-specific LLMs, uncertainty quantification of LLM annotation, and standardization of HITL backup mechanisms.

### 7.4 Theoretical boundary of the privacy–utility tradeoff

The theoretical boundary of the privacy–utility tradeoff (§3.4) has not been established: for synthetic-data generation, existing theoretical results are mostly concentrated on specific model families (e.g., convergence rates of DP-SGD, sampling-complexity lower bounds), and a tight theoretical boundary for general synthetic-data generation—given a privacy budget *ε*, what is the lower bound on synthetic-data utility—lacks a universal answer, leaving the parameter choice of privacy-preserving synthesis dependent on empirics. The future requires an information-theoretic framework for the privacy–utility tradeoff to guide parameter choice.

### 7.5 Cross-Institutional, cross-modal, and cross-disease generalization

The C4 argument notes that cross-domain generalization is the principal barrier. Federated construction (24) is constrained by data heterogeneity, multimodal fusion (46) by modality alignment, and cross-disease transfer by phenotypic differences. Under privacy constraints, source-domain data are often unavailable, making source-free domain adaptation a key path for cross-institutional generalization—the comprehensive review by Li et al. (151) surveys domain-adaptation methods when source data are unavailable. The future requires developing domain-adaptation, modality-alignment, and causal-transfer (116) methods, and establishing evaluation protocols for cross-domain generalization.

### 7.6 Verification pathways for the principal propositions

The framework's two principal propositions—C1 (closed-loop synergy) and the bias amplification chain—rest on indirect, segmented evidence rather than established results. This section gives their falsifiable verification pathways; the overall methodological boundary of the framework is in §7.8.

C1 closed-loop synergy — a proposition with a falsifiable condition. C1 hypothesizes that a complete four-pillar pipeline outperforms three-pillar pipelines missing one pillar on at least two quality dimensions without degrading a third (Table 7). This is a proposition, not an established result; a falsifiable factorial-ablation verification design is specified in Supplementary §S1.1.

**Table 7:** Unified definition of the core arguments C1–C4 of the four-pillar integration framework.

Argument	Statement	Evidence type	Boundary condition
C1 Closed-loop synergy	A complete four-pillar pipeline is hypothesized to outperform a three-pillar pipeline missing one pillar on ≥2 quality dimensions, without harming the third	Proposition: integrative derivation from independent segments (synthesis–QC in §3.6, annotation–QC in §5.3); complete factorial ablation empirics not yet available (verification pathway in §7.6)	Synthesis must have QC backing (prevent model collapse); annotation must have forward-shifted QC (intercept noise)
C2a Privacy–utility tradeoff	Stronger DP perturbation → stricter privacy guarantee but lower utility	Empirical parameter tradeoffs of DP synthesis (14, 15, 50); tight theoretical bound not established (§7.4)	Must be coupled with a formal privacy guarantee (§3.4)
C2b Bias dual-edge	Synthetic data can both amplify bias (recursive training) and debias (controlled oversampling)	Amplification: Shumailov (26); debiasing: generation-strategy empirics; overall coupling = heuristic insight (§7.6)	Amplification or debiasing depends on generation strategy + QC synergy, not synthesis *per se* (§3.6)
C3 Four-framework integration added value	Complementary organization enables cross-pillar closed-loop behaviors none of the four frameworks achieves alone	Analytical projection; the * capabilities in Table 2 await empirical verification	FAIR/ALCOA+ mechanism maturity < Metrics Reloaded/YY/T 1833; realization depends on metadata + provenance infrastructure (§7.8)
C4 Cross-domain generalization barrier	Cross-institutional, cross-modal, cross-disease generalization is the principal barrier of end-to-end pipelines	Federated: data heterogeneity (24, 25); multimodal: modality alignment (46); cross-disease: phenotypic differences; maturity gradient across 3 cases (§6.5)	Federated construction is a suboptimal choice under privacy constraints, not a free optimization (§6.2)

Bias amplification chain — established phenomena versus proposed coupling. The chain (② annotation-noise source → ① synthetic-data amplification → ③ delayed QC convergence; Figure 3) is a heuristic insight, not a necessary condition for the framework to hold. The distinction between established and proposed components is made explicit here:

- Established individual phenomena (each independently supported in the cited literature): annotation-noise propagation toward downstream degradation (Tajbakhsh (106), Karimi (107)); recursive-training model collapse as an amplification mechanism (Shumailov (26)); and the post-hoc, high-cost property of QC correction (21, 22).
- Proposed integrated mechanism (testable hypothesis, awaiting verification): that these three segments couple into a single coherent causal chain in which noise amplified through synthesis converges only at delayed QC at elevated cost—a coupling not established by the cited literature, which addresses each segment in isolation.

Falsifiable verification pathway for the proposed coupling: three-segment independent verification (propagation, amplification, and coupling arms) is specified in Supplementary §S1.2. The above constitutes a verification-design recommendation rather than an executed experiment, and the coupling awaits such empirical verification.

The QC link itself is not bias-neutral. Because forward-shifting QC can itself induce a selection bias (§5.7, including its threshold-stratification mitigation), validating the QC link as the point that terminates amplification requires confirming both that QC blocks error propagation and that its own thresholds do not introduce a countervailing selection bias. Loop 3 (deployment→upstream feedback, §6.6), being covered by the C1 factorial ablation, has similarly indirect evidentiary status.

### 7.7 Future directions: foundation-model-driven and causal-reasoning-enhanced

Two frontier directions are promising but carry characteristic risks that the closed loop must govern. Foundation-model-driven automated construction: general models represented by MedSAM (17) and medical foundation models (152) lower the annotation and synthesis barriers, making “models as data constructors” plausible—the technical foundation of four-pillar closed-loop automation; the rapid evolution of medical imaging foundation models (see Wang et al. (28)) is broadening this path. These gains are offset by seven foundation-model-specific risks: (i) hallucination (fabricated anatomical or lexical content), (ii) inherited bias (pretraining distributions propagated into medical data), (iii) domain shift (general-model representations misaligning with clinical subdomains), (iv) memorization (reproducing—and potentially leaking—training records, a privacy failure distinct from statistical fidelity), (v) overconfidence (miscalibrated certainty on out-of-distribution inputs), (vi) correlated annotation errors (systematically correlated, non-independent errors from single-model labeling that violate QC's error-independence premise), and (vii) closed-loop propagation—a foundation model's characteristic errors flow through synthesis (①) and annotation (②); if not intercepted by QC (③), they are amplified rather than damped, extending the §4.7 noise-propagation chain to model-native failure modes. Foundation-model-driven construction is therefore not an unconditional acceleration: its net benefit depends on whether the QC pillar can detect model-native errors as effectively as human-introduced ones. Causal-reasoning enhancement: the causal perspectives of Prosperi et al. (116) and Jones et al. (117) move QC from “statistical correlation” to “causal intervention,” with the potential to mitigate the bias amplification chain at its source rather than its symptoms. The two directions jointly point to automation and causalization of the four-pillar closed loop—foundation models supplying the constructive force, causal reasoning the corrective force.

### 7.8 Methodological limitations of this review

§7.1–7.5 have discussed the objective limitations of the field; this section supplements the inherent boundaries of the methodology. This is a narrative review, with reporting quality following SANRA (Baethge et al. (153)); it does not adopt PRISMA, because it lacks the prospective registration, exhaustive retrieval, and dual-reviewer screening of a systematic review.

The four pillars and the four categories of enabling technologies in §1.3 derive from the same literature; the closed-loop organizational property is an analytical derivation rather than an independent empirical verification—an inherent constraint of a narrative review.

Exhaustive systematic retrieval was not conducted; representative literature (2014–2025) was located via iterative PubMed and IEEE Xplore queries seeded by concept terms spanning the four pillars—synthetic data (GAN, diffusion, differential privacy, EHR), intelligent annotation (active learning, semi-/weak supervision, LLM annotation, vision–language models, foundation models), automated quality control (metrics, label noise, bias detection; compliance: YY/T 1833, FDA GMLP, EU AI Act), and integration (federated learning, multimodal fusion, nnU-Net, MONAI)—plus citation chasing and domain knowledge that also located competing conceptual frameworks. The 156 references are a representative rather than exhaustive set (peer-reviewed papers, public standards, tools, and public datasets; no preprints). The Table 1 judgment that existing reviews each cover a single pillar and do not characterize the four-pillar closed-loop relationship is a negative assertion bounded by the included literature. The bias-assessment checklist has not been pre-test calibrated.

### 7.9 Conclusion

This review proposes a four-pillar integration framework (Figure 1)—synthetic data generation, intelligent annotation, automated quality control, and integration pipelines, organized as a closed loop with dual-track synthesis–annotation feedback and bidirectional annotation–QC verification—making cross-pillar emergent relationships explicit. Synthesizing the four research questions: the method spectrum has evolved from GANs and manual annotation toward diffusion, LLM, and foundation models (RQ1); these methods approach real data in statistical fidelity and downstream utility for select modalities, tasks, and evaluation settings, but exhibit systematic gaps in privacy, fairness, and clinical validity (RQ2); the framework extends Metrics Reloaded, FAIR, ALCOA+, and YY/T 1833 as complementary sub-layer evaluation dimensions (RQ3); and integration pipelines are usable for single-disease single-modality cases while cross-domain generalization remains the principal barrier (RQ4). Future work should advance foundation-model-driven automated construction and causal-reasoning enhancement, driving the four-pillar closed loop toward automation and causalization, so that medical dataset quality enhancement can mature from single-point techniques to systems engineering and move medical AI from narrow-task performance toward trustworthy clinical deployment.

### 7.10 Contribution to the field

Artificial intelligence is increasingly used in medicine, but its success depends on the quality of the datasets used to train it. Building medical datasets by hand is slow, expensive, and prone to gaps and bias, while the methods meant to improve these datasets have so far been studied one at a time and in isolation. This review brings four families of methods—generating synthetic data, intelligent annotation, automated quality checking, and integration pipelines—together into a single closed-loop framework, showing how they feed back into one another rather than working as separate steps. By organizing the scattered evidence in this way, the review makes relationships visible that no single-method study can show on its own, including a chain through which bias can be amplified when these techniques are combined without oversight. Compared with existing reviews that each cover only one technique, the main contribution is this integrated, closed-loop view, which offers researchers and developers a shared map for building higher-quality medical datasets and points to where the field most needs validation and standards.
