# Supplementary material for: AI-driven quality enhancement of medical datasets: a closed-loop framework of synthesis, annotation, quality control, and integration
Source: Front Med (Lausanne). 2026 Oct 1;13:1947584. doi: 10.3389/fmed.2026.1947584 (PMC13669839; doi:10.3389/fmed.2026.1947584)
Supplement: Supplementary file 1 [file Datasheet1.docx]

**Supplementary Methodological Detail**

This supplementary file accompanies the manuscript "AI-Driven Quality Enhancement of Medical Datasets: A Closed-Loop Framework of Synthesis, Annotation, Quality Control, and Integration." It consolidates methodological specifications, detailed enumeration of risks, and verification-design recommendations. All reference numbers correspond to the main-text reference list [1]–[156]; no new references are introduced here.

# **S1. Falsifiable Verification-Design Specification for the Core Propositions**

## **S1.1 C1 closed-loop synergy — proposed factorial ablation pathway**

C1 hypothesizes that a complete four-pillar pipeline outperforms three-pillar pipelines missing one pillar on at least two quality dimensions without degrading a third (main-text Table 3). Because comparing pipelines with and without QC isolates only the QC contribution and cannot establish synergy among all four pillars, verification requires a factorial ablation design.

Design. A 2⁴ full-factorial design over the four pillars (presence/absence, yielding 16 pipeline configurations including the empty baseline), evaluated across nine dimensions:

1. Fidelity

2. Annotation accuracy

3. Downstream performance

4. Calibration

5. Subgroup performance

6. Privacy

7. Representativeness

8. Robustness

9. Computational cost

Benchmark cases. The three benchmark cases of main-text §6.5 (lung nodule, diabetic retinopathy, pathology WSI), with confounders—synthesis fidelity, privacy budget, and annotation budget—controlled across configurations.

Falsifiable condition. The complete four-pillar configuration must outperform every three-pillar configuration on at least two quality dimensions without degrading any third. Should some three-pillar configurations match or exceed the complete configuration, the closed-loop property would be non-essential and C1 falsified for that setting.

Status. This is a verification-design recommendation for subsequent empirical research, not executed work; until relevant empirics appear, C1 remains a proposition.

## **S1.2 Falsifiable verification pathway for the proposed bias-amplification coupling**

The bias amplification chain (② annotation-noise source → ① synthetic-data amplification → ③ delayed QC convergence; main-text Figure 3) is a heuristic insight whose three segments are each independently supported but whose coupling into a single causal mechanism awaits verification. Three-segment independent verification is proposed:

1. Propagation segment. Inject annotation noise of a known distribution on a controlled benchmark and measure the dose–response relationship between downstream degradation and noise intensity.

2. Amplification segment. Compare degradation rates between real-data and synthetic-data recursive training, testing whether the model-collapse irreversibility established by Shumailov [20] holds in medical scenarios.

3. Coupling segment (most critical). A three-arm experiment—no QC / post-hoc QC / forward-shifted QC—testing whether forward-shifting QC blocks the entire chain and reverses downstream degradation.

The above is a verification-design recommendation rather than an executed experiment. Until relevant empirics appear, the bias amplification chain should be treated as a heuristic insight with independent segmented-evidence support but whose overall coupling awaits verification, not a proven mechanism.

# **S2. Missingness Mechanisms and Fusion Risks in Multimodal Data**

The challenges of multimodal fusion extend beyond modality alignment to the mechanisms by which modalities go missing. Missingness is not uniform: under MCAR (missing completely at random) loss is unrelated to any variable and imputation remains unbiased; under MAR (missing at random) loss depends on observed variables and is conditionally addressable; under MNAR (missing not at random) loss depends on the unobserved value itself, producing systematic bias that imputation cannot fully correct. MNAR is the most severe mechanism because it silently encodes the absence of exactly the hardest-to-represent cases, and multimodal datasets are particularly vulnerable because a single missing modality can cascade into degraded joint representations.

Beyond missingness, four further risks shape multimodal dataset validity: temporal misalignment (modalities sampled at inconsistent timescales blur the physiological state they purport to capture jointly); patient-level linkage errors (incorrect matching of imaging, reports, and EHR across systems injects label noise at the fusion boundary rather than within a single modality); modality-specific measurement error (each modality carries its own noise floor, and fusion propagates rather than cancels these errors when modalities are correlated); and information leakage (a multimodal joint record is often easier to re-identify than any single unimodal record, so multimodal integration can reduce rather than enhance privacy).

# **S3. Pathology WSI Quality Challenges**

Computational pathology presents several quality challenges that are especially acute for AI development and distinct from radiology and ophthalmology: scanner and staining variability (whole-slide appearance shifts across scanner vendors and staining protocols, producing substantial cross-institutional domain shift); tissue-processing artifacts (fixation, sectioning, and coverslipping introduce artifacts that are neither label nor noise but confounders); multi-scale slide/tile relationships (a gigapixel slide is tiled for computation, but tile-level labels and slide-level labels are related yet not equivalent, so aggregation across scales is itself a QC problem); slide-level versus region-level annotation (pathologist Region-of-Interest annotation does not map cleanly to pixel-level labels, and weak supervision from slide-level reports is common but lossy); inter-observer variability (diagnostic concordance among pathologists is historically lower than among radiologists for many tasks, raising the floor on achievable label noise); and diagnostic label provenance (labels derived from differing grading schemes and molecular definitions are not interchangeable across cohorts). These challenges make QC at gigapixel scale particularly demanding—slide/tile consistency checking is not a minor extension of radiology QC but a distinct problem.

It is important to distinguish MedMNIST/PathMNIST [153] from true gigapixel WSI datasets: PathMNIST provides pre-tiled, pre-labeled 2D image patches at fixed resolution, which is valuable as a lightweight benchmark but bypasses the slide/tile relationship, scanner variability, and region-level annotation problems that define WSI dataset construction. Patch-level benchmark datasets and whole-slide datasets therefore represent substantially different data-construction and QC challenges and should not be treated as interchangeable.
